# Cramér–Rao Lower Bound for Magnetic Field Localization around Elementary Structures

**DOI:** 10.3390/s24082402

**Published:** 2024-04-09

**Authors:** Armin Dammann, Benjamin Siebler, Stephan Sand

**Affiliations:** German Aerospace Center (DLR), Oberpfaffenhofen, 82234 Wessling, Germany; benjamin.siebler@DLR.de (B.S.); stephan.sand@DLR.de (S.S.)

**Keywords:** navigation, position estimation, estimation theory, magnetic field, magnetic sensor, estimation error, Fisher information, Cramér–Rao lower bound

## Abstract

The determination of a mobile terminal’s position with high accuracy and ubiquitous coverage is still challenging. Global satellite navigation systems (GNSSs) provide sufficient accuracy in areas with a clear view to the sky. For GNSS-denied environments like indoors, complementary positioning technologies are required. A promising approach is to use the Earth’s magnetic field for positioning. In open areas, the Earth’s magnetic field is almost homogeneous, which makes it possible to determine the orientation of a mobile device using a compass. In more complex environments like indoors, ferromagnetic materials cause distortions of the Earth’s magnetic field. A compass usually fails in such areas. However, these magnetic distortions are location dependent and therefore can be used for positioning. In this paper, we investigate the influence of elementary structures, in particular a sphere and a cylinder, on the achievable accuracy of magnetic positioning methods. In a first step, we analytically calculate the magnetic field around a sphere and a cylinder in an outer homogeneous magnetic field. Assuming a noisy magnetic field sensor, we investigate the achievable positioning accuracy when observing these resulting fields. For our analysis, we calculate the Cramér–Rao lower bound, which is a fundamental lower bound on the variance of an unbiased estimator. The results of our investigations show the dependency of the positioning error variance on the magnetic sensor properties, in particular the sensor noise variance and the material properties, i.e., the relative permeability of the sphere with respect to the cylinder and the location of the sensor relative to the sphere with respect to the cylinder. The insights provided in this work make it possible to evaluate experimental results from a theoretical perspective.

## 1. Introduction

Position information is becoming increasingly important in mobile communication systems. Location-based services and navigation applications require such position information with sufficient accuracy and availability in both outdoor and indoor environments. In addition to applications, lower layers in mobile communication systems can benefit from information about the mobile terminals’ locations as well [1].

There are a variety of technologies for the determination of a mobile terminal’s position. Global navigation satellite systems [2] like GPS, GLONASS, Galileo, Beidou, etc. provide good accuracy and coverage in outdoor areas. Indoors, however, non-line-of-sight and multipath propagation, together with weak signal power levels at the receiver, make satellite-based positioning challenging or even impossible. In such challenging environments, positioning based on terrestrial wireless networks is considered to be the complementary technology. Received signals in a terrestrial wireless network provide a relation between the position of a mobile terminal and the positions of the base stations in the network. Several characteristics of a received signal carry information about the position of a mobile terminal [3]. The time of arrival (TOA) and time difference of arrival (TDOA) exploit the signal propagation delay between base stations and the mobile terminal for positioning. Other methods make use of the angle of arrival (AOA) or the received signal strength (RSS) for mobile terminal positioning. Compared to satellite signals, terrestrial mobile radio signals provide higher power levels at the receiver. As in the case of satellite-based positioning, multipath and non-line-of-sight propagation also degrade the positioning performance based on terrestrial wireless networks severely. Furthermore, at least three base stations must be observable to a mobile terminal in order to obtain a two-dimensional position fix. This is hardly achieved in today’s mobile radio networks. In the future, this situation might either change due to denser network cell structures, or the requirement of receiving multiple base stations will become obsolete by applying cooperative positioning methods [4].

Especially for pedestrian indoor navigation, the use of inertial sensors became popular in recent years. Such sensors are cheap and widely deployed in today’s mobile devices. However, if conventional dead reckoning is applied using inertial sensors, the positioning error grows cubically in time due to sensor drift. In [5], the author proposes a pedestrian tracking system using a foot-mounted inertial sensor. By detecting the resting phase of the foot during a typical human walk, so-called zero-velocity updates are applied, which reduce the positioning error from cubic to linear. However, the problem of an unlimited growth of the positioning error remains. A step forward for solving the drift problem is to use landmarks, i.e., location-dependent characteristics that can be observed and identified. By regularly revisiting such landmarks, remaining inertial sensor drifts can be compensated. Mobile radio networks or dedicated positioning systems can provide such landmarks. To achieve an appropriate local distribution of landmarks, i.e., reference or base stations, some installation of additional infrastructure might be required. Additionally, this infrastructure has to be calibrated so that the positions of these reference stations are known. Both the installation and calibration of positioning infrastructure is time-consuming and costly. Therefore, a common approach is to use landmarks that can be detected with today’s mobile terminal low-cost sensor equipment. Using the principles of simultaneous localization and mapping (SLAM), the positions of the mobile terminal and the landmarks are estimated at the same time, i.e., calibration happens on the fly. In [6], the authors propose a pedestrian navigation system that combines odometry, obtained from a foot-mounted inertial sensor, and RSS measurements of WiFi base stations as landmarks. Both the mobile terminal and the WiFi bass stations’ positions are estimated using a SLAM approach based on Bayesian filtering. The authors of [7] introduce an indoor positioning principle, solely based on a foot mounted inertial sensor, called FootSLAM. The structure of walking routes are used as location-dependent characteristics. The FootSLAM algorithm method detects and maps these walking routes. When revisited, mapped walking routes are recognized that are used for the compensation of the inertial sensor drift.

Another kind of location-dependent characteristic that can be used for positioning is the Earth’s magnetic field. Ferromagnetic materials placed in that field cause distortions. Regardless if being distorted or not, the Earth’s magnetic field observed by a magnetic sensor in a mobile device in general depends on the position and/or the orientation of the mobile device. A variety of positioning methods based on the observation of the Earth’s magnetic field have been proposed and studied, which relate to our investigations. Many of these methods come with experimental validation. This paper aims to provide basic theoretical dependencies for magnetic field-based positioning so that the experimental results regarding positioning performance can ultimately be evaluated and explained from a theoretical perspective.

### 1.1. Related Work

The discovery and observation of magnetic phenomena goes back even before the common era. As one result, the compass had already been developed centuries ago [8]. A compass indicates the direction of a surrounding magnetic field. In open areas, the Earth’s magnetic field provides orientation towards the magnetic poles of the Earth. More complex environments like indoors show severe distortions of the Earth’s magnetic field, which are mainly caused by surrounding ferromagnetic materials such as reinforcing steel, for instance. A compass usually fails in such areas. Such distortions, however, are location-dependent characteristics and can be considered as a local magnetic fingerprint.

In [9], the authors study the feasibility of using such fingerprints for positioning. They found that the Earth’s magnetic field is temporarily stable enough, and its distortions can be sufficiently significant in order to achieve submeter or even down to decimeter positioning accuracy with fingerprinting methods. Obviously, higher local distortions in the magnetic field provide higher positioning accuracy. Fingerprinting positioning methods require a database, which must be created in a training or calibration phase.

Investigations in [10] address the behavior of the Earth’s magnetic field in the vicinity of ferromagnetic materials. It was shown that ferromagnetic objects like pillars can be identified and used as landmarks by observing significant changes in the magnetic field strength around them. Furthermore, the experiments described in [10] show a good match between the measured magnetic field strength and its prediction by analytical approximations. Also, long-term stability and reproducibility of the magnetic field observations are found to be suitable for magnetic fingerprinting positioning.

In [11], Angermann et al. proposed to exploit the characteristic Earth magnetic field perturbations for indoor positioning. The authors discuss various methods for the measurement and mapping of the Earth’s magnetic field with sufficient granularity. For repeated magnetic field measurements, only little noise was identified, which indicated good stability and reproducibility of the Earth magnetic field observations. An autonomous robotic platform for high resolution and complete mapping of the Earth’s magnetic field has been developed and described in [12].

The suitability of the magnetic field was also shown for road [13] and railway environments [14]. Even in the airspace, the magnetic field shows distortions that, in combination with an inertial navigation system, can be exploited for position estimation [15]. Unlike in the other environments, the distortions in the airspace are not caused by nearby magnetic material but by the crustal field of the Earth [15], which typically is smaller than the distortions observed, e.g., in indoor environments.

In [16], the theoretically achievable accuracy of magnetic localization for a wheeled robot driving through an indoor environment has been analyzed by deriving a Bayesian lower bound. The bound was based on a Gaussian process fitted to a data set of densely measured magnetic field vectors. Thus, the bound requires an exhaustive measurement campaign to be calculated, but once the data set is available, the bound can be used to assess the quality of different filter algorithms. The paper showed that particle filters perform well but are not optimal with respect to to their mean square error.

In contrast to performing magnetic field mapping as a required calibration step prior to solving the actual positioning task, several research works propose using SLAM. In [17], the authors provide an experimental proof of a concept for a SLAM approach that uses the Earth’s magnetic field anomalies in combination with the odometry data of a robotic platform. The magnetic field there was parametrically modeled using Gaussian processes. The combination of magnetic and inertial sensors in a SLAM-based positioning solution has been proposed in [18,19,20]. With this sensor combination, the authors of [19] obtained positioning errors in the order of 10 cm to 20 cm when walking through a large building.

The works and methods mentioned above utilize the distortions of the Earth’s magnetic field for positioning. They mainly rely on experimental work for characterizing and mapping the Earth’s magnetic field and its perturbations for positioning—either with a traditional calibration step prior to running positioning algorithms or with SLAM-based solutions. In order to quantify the positioning performance, estimated positions obtained during experiments have been compared to the corresponding ground truth. Our work presented in this paper complements these results by analyzing the achievable positional accuracy using an estimation–theoretic approach based on the computation of Fisher information and the Cramér–Rao lower bound.

### 1.2. Contribution

In this paper, we aim to lower bound the achievable error variance for positioning methods based on the observation of the ambient magnetic field. In particular, we address the following questions:

- How does the achievable positioning performance depend on the magnetic sensor properties, i.e., the sensor noise variance?
- How does the positioning performance depend on the distance from ferromagnetic objects that cause magnetic field distortions?
- How does the ferromagnetic material properties, in particular the relative permeability, influence the achievable positioning performance?

To answer these questions, we provide an analytical description of the magnetic field around a sphere and a cylinder placed in an outer homogeneous static magnetic field in Section 2 and Section 3. We use these analytical field descriptions and calculate the Cramér–Rao lower bound for position estimation based on noisy magnetic field observations in Section 4. Section 5 provides examples and evaluations of the achievable positioning performance for a ferromagnetic cylinder in the Earth’s magnetic field.

## 2. The Static Magnetic Field

Maxwell’s Equations—a set of four partial differential equations—describe the behavior and interrelation of charges, currents, and electric and magnetic fields. According to these equations, dynamic electric and magnetic fields influence each other through nonzero partial derivatives of those fields with respect to time. In the static case, however, these derivatives are zero, and Maxwell’s system of equations decouples. So, we obtain two equations—in particular the Maxwell–Ampère equation and Gauss’s law for magnetism—which describe any static magnetic field.

### 2.1. Maxwell’s Equations for the Static Magnetic Field

Maxwell’s equations for the static magnetic field can be found in many textbooks on electromagnetic field theory, e.g., in [21,22]. The Maxwell–Ampère equation for the static magnetic strength vector field H in the absence of currents reads as

(1) rotH=0.

Another equation, describing the magnetic field, is Gauss’s law for magnetism. It states that the magnetic flux density vector field is defined as

(2) divB=divμH=0,

with B=μH being is a divergence-free vector field. In other words, this means that there are no magnetic charges. Here, μ is a constant describing the material’s permeability. With these conditions, we can express the magnetic strength vector field

(3) H=−gradΨ=−∇Ψ

as the gradient of a scalar potential field Ψ. This choice fulfills (1), since the curl of a gradient field is zero in general. Substituting (3) into (2) yields Laplace’s equation:

(4) divgradΨ=ΔΨ=0.

This partial differential equation usually comes with boundary conditions. Thus, a solution to that boundary value problem solves Laplace’s equation and additionally satisfies the boundary conditions.

### 2.2. Boundary Values and Conditions

As mentioned above, we have to find a scalar magnetic potential field that is a solution of Laplace’s equation and satisfies given boundary values and conditions. General boundary value constraints are the continuity of the parallel magnetic strength vector components $H_{i}^{\|}$ and the perpendicular magnetic flux density vector components $B_{i}^{⊥}$ at the boundary surfaces between materials, indexed by *i*, with different permeabilities, i.e.,

(5) $$\begin{array}{ccc} H_{1}^{\|} & = & H_{2}^{\|}, \end{array}$$

(6) $$\begin{array}{ccc} B_{1}^{⊥} & = & B_{2}^{⊥}. \end{array}$$

The boundary condition for the magnetic field strength components (5) follow from the Maxwell–Ampère Equation (1). The continuity of the perpendicular magnetic flux density (6) is a consequence of Gauss’s law for magnetism (2).

Subsequently, we investigate the magnetic field that results from the presence of a ferromagnetic body like an iron sphere or cylinder in an outer magnetic field. It is reasonable to assume that the influence of the ferromagnetic body on the magnetic field decays with increasing distance. This yields the boundary value at infinite distance to the ferromagnetic body in such a way that the solution of the boundary value problem, −∇Ψ, converges to the original field $H_{\infty }$, which would be observable in absence of the ferromagnetic structure. This assumption leads to the Neumann boundary condition:

(7) $$\lim_{r\to \infty }-\nabla \Psi =H_{\infty }.$$

Equivalently, we can require the magnetic potential field to take on specific values. In this case, we obtain the Dirichlet boundary condition

(8) $$\lim_{r\to \infty }\Psi =\Psi_{\infty },$$

which, except for an additive constant, is equivalent to the Neumann boundary condition.

The usual procedure of solution for boundary value problems is to find a general solution for Laplace’s equation for each volume having a different permeability. Using their remaining degrees of freedom, these solutions are then aligned to each other in order to meet the given boundary values and constraints.

## 3. Ferromagnetic Structures in a Homogeneous Magnetic Field

As mentioned earlier, ferromagnetic materials cause distortions in the Earth’s ambient magnetic field, which are inherently location dependent and can thus be used for position estimation. In daily life, there are many environments, like indoors, where we find ferromagnetic materials in close proximity. Just think of the reinforcement steel in the concrete hulls of buildings as an example.

In the following sections, we provide an analytical description of the magnetic potential field or magnetic field strength for a sphere and a cylinder, respectively, which we place into an external homogeneous magnetic field.

### 3.1. Sphere

We consider an outer homogeneous magnetic strength vector field

(9) $$H_{\infty }=H_{\infty }\;e_{z}=H_{\infty }\cos\left(\theta \right)\;e_{r}-H_{\infty }\sin\left(\theta \right)\;e_{\theta }$$

with magnitude $H_{\infty }$, which is oriented in *z* direction of a Cartesian coordinate system. The second part of (9) provides the description of this field in spherical coordinates with unit vectors $e_{r}$ in the radial direction and $e_{\theta }$ in the polar direction. This static magnetic field can be obtained from the corresponding scalar magnetic potential field:

(10) $$\Psi_{\infty }=-H_{\infty }\;z=-H_{\infty }\;r\;\cos\left(\theta \right).$$

**Figure 1:**
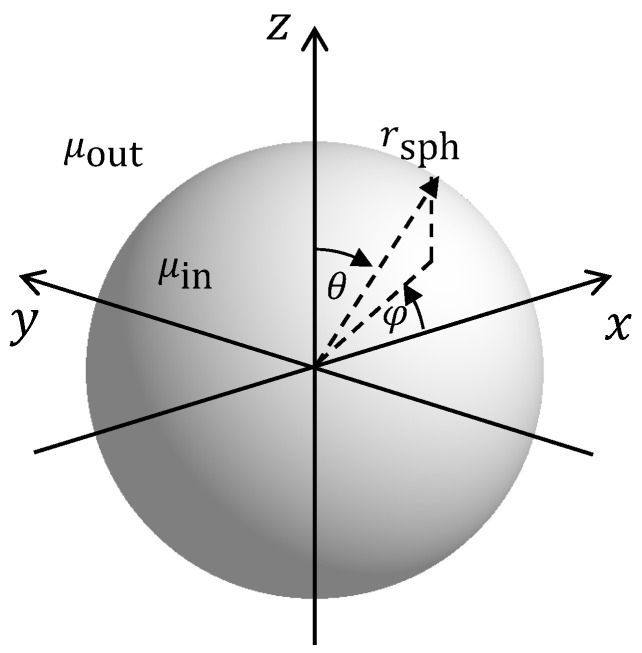
Solid sphere with permeability $\mu_{\mathrm{in}}$ in an outer medium with permeability $\mu_{\mathrm{out}}$.

Now, let us place a sphere with a permeability $\mu_{\mathrm{in}}$ and a radius $r_{\mathrm{sph}}$ into this magnetic field at the origin of the coordinate system, as shown in Figure 1.

The resulting magnetic field can be calculated by solving Laplace’s Equation (4) for the magnetic scalar potential field. We split this field into the magnetic potential field $\Psi_{\mathrm{in}}$ within the sphere and the outer field $\Psi_{\mathrm{out}}$. At the sphere’s surface, the boundary conditions (5) and (6) must hold. Additionally, the outer field has to fulfill the boundary condition $\lim_{r\to \infty }\Psi_{\mathrm{out}}=\Psi_{\infty }$.

For solving Laplace boundary value problems, a common approach is to apply the ‘separation of variables’ method. There, we assume a factorized solution of Ψ(r,φ,θ)=R(r)Φ(φ)Θ(θ), where each of the three factors only depends on one coordinate. This approach separates the Laplace differential equation into three ordinary differential equations—one for each factor. These ordinary differential equations are easier to solve. The solution of the Laplace boundary value problem through the separation of variables is well known and therefore not in the focus of this paper. Interested readers are referred to textbooks, e.g., ([21], p. 247 f.) or Appendix B. At the end, we obtain a solution of the Laplace equation for the outer magnetic potential field of our problem as

(11) $$\Psi_{\mathrm{out}}=H_{\infty }\left(\frac{\frac{\mu_{\mathrm{in}}}{\mu_{\mathrm{out}}}-1}{\frac{\mu_{\mathrm{in}}}{\mu_{\mathrm{out}}}+2}{\left(\frac{r_{\mathrm{sph}}}{r}\right)}^{3}-1\right)\;r\;\cos\left(\theta \right).$$

Correspondingly, the inner magnetic potential field is

(12) $$\Psi_{\mathrm{in}}=-H_{\infty }\;\;\frac{3}{\frac{\mu_{\mathrm{in}}}{\mu_{\mathrm{out}}}+2}\;r\;\cos\left(\theta \right).$$

With the gradient operator $\nabla :=\left(\frac{\partial }{\partial r},\frac{1}{r}\frac{\partial }{\partial \theta },\frac{1}{r\;\sin(\theta )}\frac{\partial }{\partial \varphi }\right)$ for spherical coordinates, we obtain the corresponding magnetic field strengths as

(13) $$\begin{array}{cc} H_{\mathrm{out}}=-\nabla \;\Psi_{\mathrm{out}}= & H_{\infty }\left(2\;\frac{\mu_{r}-1}{\mu_{r}+2}{\left(\frac{r_{\mathrm{sph}}}{r}\right)}^{3}+1\right)\;\cos\left(\theta \right)\;e_{r}\\ & +H_{\infty }\left(\frac{\mu_{r}-1}{\mu_{r}+2}{\left(\frac{r_{\mathrm{sph}}}{r}\right)}^{3}-1\right)\;\sin\left(\theta \right)\;e_{\theta } \end{array}$$

and

(14) $$\begin{array}{cc} H_{\mathrm{in}}=-\nabla \;\Psi_{\mathrm{in}}= & H_{\infty }\;\frac{3}{\mu_{r}+2}\;\cos\left(\theta \right)\;e_{r}\\ & -H_{\infty }\;\frac{3}{\mu_{r}+2}\;\sin\left(\theta \right)\;e_{\theta }. \end{array}$$

For convenience, we have set $\mu_{r}=\frac{\mu_{\mathrm{in}}}{\mu_{\mathrm{out}}}$. It is straightforward to verify this solution by insertion into (4)–(8). For further investigations, we will require the magnetic strength vector field outside the sphere in Cartesian coordinates. From (13) and with

(15) $$\begin{array}{ccc} e_{r} & = & \sin\left(\theta \right)\cos\left(\varphi \right)\;e_{x}+\sin\left(\theta \right)\sin\left(\varphi \right)\;e_{y}+\cos\left(\theta \right)\;e_{z} \end{array}$$

(16) $$\begin{array}{ccc} e_{\theta } & = & \cos\left(\theta \right)\cos\left(\varphi \right)\;e_{x}+\cos\left(\theta \right)\sin\left(\varphi \right)\;e_{y}-\sin\left(\theta \right)\;e_{z} \end{array}$$

we obtain

(17) $$\begin{array}{cc} H_{\mathrm{out}}= & \frac{3}{2}H_{\infty }\;\frac{\mu_{r}-1}{\mu_{r}+2}{\left(\frac{r_{\mathrm{sph}}}{r}\right)}^{3}\;\sin\left(2\;\theta \right)\;\cos\left(\varphi \right)\;e_{x}\\ & +\frac{3}{2}H_{\infty }\;\frac{\mu_{r}-1}{\mu_{r}+2}{\left(\frac{r_{\mathrm{sph}}}{r}\right)}^{3}\;\sin\left(2\;\theta \right)\;\sin\left(\varphi \right)\;e_{y}\\ & +\frac{1}{2}H_{\infty }\left[2+\frac{\mu_{r}-1}{\mu_{r}+2}{\left(\frac{r_{\mathrm{sph}}}{r}\right)}^{3}\;\left(3\;\cos(2\;\theta )+1\right)\right]\;e_{z}. \end{array}$$

**Figure 2:**
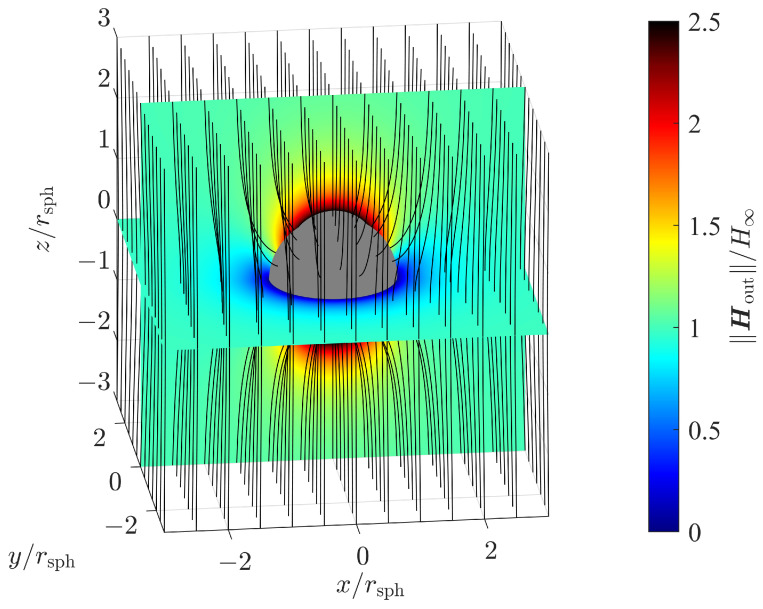
Magnetic field strength and magnetic flux lines for a sphere with relative permeability $\mu_{r}=100$ in an outer homogeneous magnetic field.

Figure 2 shows the magnetic field strength for a ferromagnetic sphere with a relative permeability of $\mu_{r}=\frac{\mu_{\mathrm{in}}}{\mu_{\mathrm{out}}}=100$ as a color plot exemplary for slice planes y=0 and z=0. The magnetic field strength is expressed by its magnitude normalized to the homogeneous field strength $H_{\infty }$. Its orientation is shown by magnetic flux lines.

### 3.2. Cylinder

**Figure 3:**
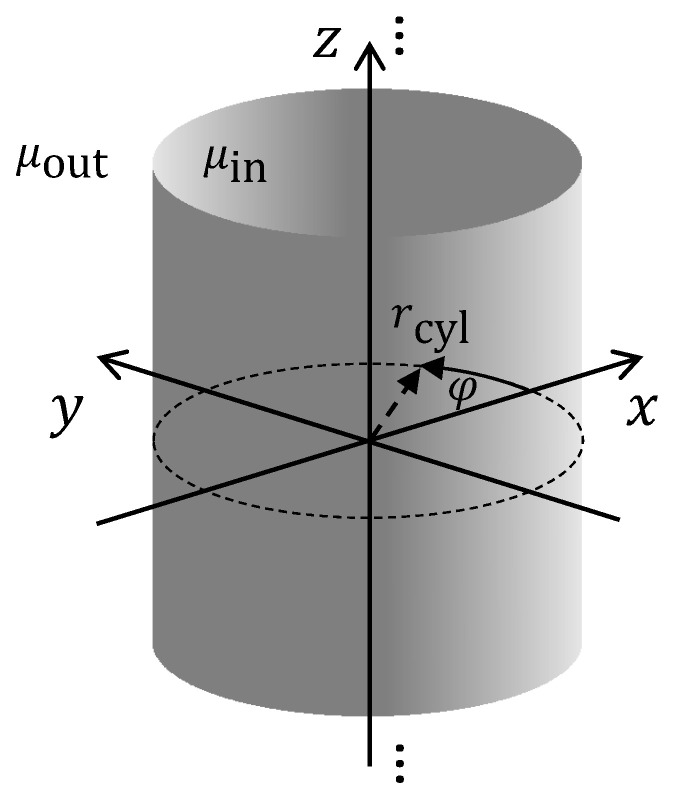
Solid cylinder with permeability $\mu_{\mathrm{in}}$ in an outer medium with permeability $\mu_{\mathrm{out}}$.

We assume a cylinder of infinite length with a permeability $\mu_{\mathrm{in}}$ and a radius $r_{\mathrm{sph}}$ located along the z axis of a cylindrical coordinate system, as shown in Figure 3.

Due to its permeability $\mu_{\mathrm{in}}$ being different from that of the outer medium $\mu_{\mathrm{out}}$, this cylinder influences an outer homogeneous magnetic field. First, let us consider a homogeneous outer magnetic field with arbitrary direction. Since the dimension of the cylinder in the *z* direction is infinite, there is no dependency of the resulting magnetic field on dimension *z*. For this reason, it is obvious that the cylinder does not influence the *z* component of an outer homogeneous magnetic field. Therefore, we consider a homogeneous outer magnetic field strength with its direction in the *x*–*y* plane. Without loss of generality, we assume a homogeneous magnetic strength vector field in the *x* direction with magnitude $H_{\infty }$, which we can express as

(18) $$H_{\infty }=H_{\infty }\;e_{x}.$$

The resulting magnetic field can be obtained by solving Laplace’s Equation (4) for the magnetic scalar potential field. We split this field into the magnetic potential field $\Psi_{\mathrm{in}}$ within the cylinder and the outer field $\Psi_{\mathrm{out}}$. The outer field has to fulfill the boundary condition $-\mathrm{grad}\Psi_{\mathrm{out}}=H_{\infty }\;e_{x},\;r\to \infty$. The solution for the outer field is

(19) $$\Psi_{\mathrm{out}}=H_{\infty }\left(\frac{\frac{\mu_{\mathrm{in}}}{\mu_{\mathrm{out}}}-1}{\frac{\mu_{\mathrm{in}}}{\mu_{\mathrm{out}}}+1}{\left(\frac{r_{\mathrm{cyl}}}{r}\right)}^{2}-1\right)\;r\;\cos\left(\varphi \right).$$

Correspondingly, the inner magnetic potential field is

(20) $$\Psi_{\mathrm{in}}=-H_{\infty }\;\;\frac{2}{\frac{\mu_{\mathrm{in}}}{\mu_{\mathrm{out}}}+1}\;r\;\cos\left(\varphi \right).$$

A derivation for this solution can be found in Appendix C. With the gradient operator $\nabla :=\left(\frac{\partial }{\partial r},\frac{1}{r}\frac{\partial }{\partial \varphi },\frac{\partial }{\partial z}\right)$ for cylindrical coordinates, we obtain the corresponding magnetic field strengths as

(21) $$\begin{array}{cc} H_{\mathrm{out}}=-\nabla \Psi_{\mathrm{out}}= & H_{\infty }\;\left(\frac{\mu_{r}-1}{\mu_{r}+1}\;{\left(\frac{r_{\mathrm{cyl}}}{r}\right)}^{2}+1\right)\;\cos\left(\varphi \right)\;e_{r}\\ & +H_{\infty }\;\left(\frac{\mu_{r}-1}{\mu_{r}+1}\;{\left(\frac{r_{\mathrm{cyl}}}{r}\right)}^{2}-1\right)\;\sin\left(\varphi \right)\;e_{\varphi } \end{array}$$

outside the cylinder and

(22) $$\begin{array}{cc} H_{\mathrm{in}}=-\nabla \Psi_{\mathrm{in}}= & H_{\infty }\;\frac{2}{\mu_{r}+1}\;\cos\left(\varphi \right)\;e_{r}\\ & -H_{\infty }\;\frac{2}{\mu_{r}+1}\;\sin\left(\varphi \right)\;e_{\varphi } \end{array}$$

inside the cylinder. For convenience, we have set $\mu_{r}=\frac{\mu_{\mathrm{in}}}{\mu_{\mathrm{out}}}$. For further investigations, we express the magnetic strength vector field in Cartesian coordinates. From (21) and with

(23) $$\begin{array}{ccc} e_{r} & = & \cos\left(\varphi \right)\;e_{x}+\sin\left(\varphi \right)\;e_{y} \end{array}$$

(24) $$\begin{array}{ccc} e_{\varphi } & = & -\sin\left(\varphi \right)\;e_{x}+\cos\left(\varphi \right)\;e_{y} \end{array}$$

we obtain

(25) $$\begin{array}{cc} H_{\mathrm{out}}= & H_{\infty }\;\left(1+\frac{\mu_{r}-1}{\mu_{r}+1}\;{\left(\frac{r_{\mathrm{cyl}}}{r}\right)}^{2}\;\cos\left(2\;\varphi \right)\right)\;e_{x}\\ & +H_{\infty }\;\frac{\mu_{r}-1}{\mu_{r}+1}\;{\left(\frac{r_{\mathrm{cyl}}}{r}\right)}^{2}\;\sin\left(2\;\varphi \right)\;e_{y}. \end{array}$$

**Figure 4:**
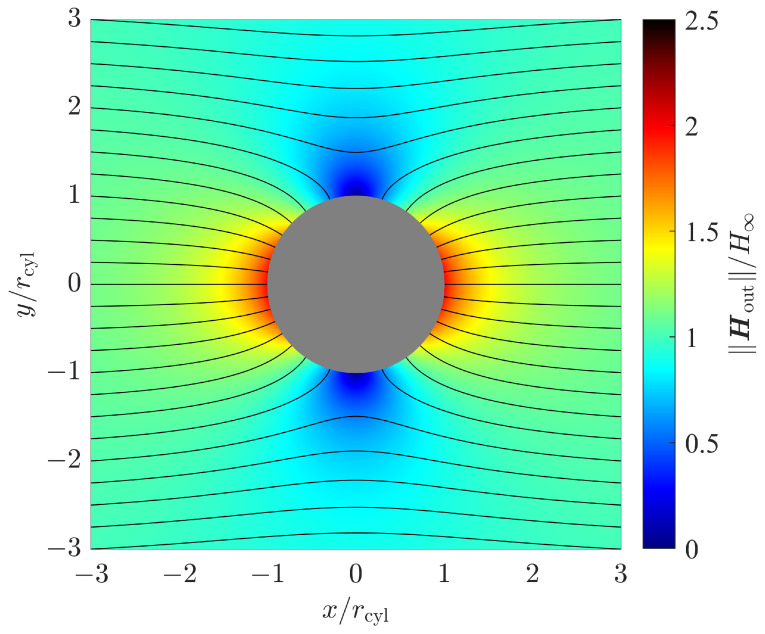
Magnetic field strength and magnetic flux lines for a cylinder with relative permeability $\mu_{r}=100$ in an outer homogeneous magnetic field.

Figure 4 shows the magnetic field strength for a ferromagnetic sphere with a relative permeability of $\mu_{r}=\frac{\mu_{\mathrm{in}}}{\mu_{\mathrm{out}}}=100$. The magnetic field strength is expressed by its magnitude normalized to the homogeneous field strength $H_{\infty }$. Its orientation is shown by magnetic flux lines. Since the magnetic field components neither contain components in the *z* direction nor show a dependency on the *z* coordinate, it is sufficient to plot the magnetic field in two dimensions, i.e., in the *x*–*y* plane with z=0.

## 4. Positioning Performance—An Estimation–Theoretic Approach

Previously, we have calculated the magnetic field strength when placing elementary structures, in particular a cylinder and a sphere, in an outer homogeneous magnetic field. These magnetic fields $H\left(a\right)$ depend on a variety of parameters, which we collect in a vector $a=\left(a_{1},\ldots ,a_{N}\right)$. Since the magnetic field is location dependent, the parameter vector a also contains location coordinates x. Thus, we can infer position information from the magnetic field strength measurement values. Usually, such measurements are noisy, which causes noisy position estimates. We are interested in the achievable performance of positioning based on noisy magnetic field strength measurements, in particular the variance in the noisy position estimates.

For the evaluation of the position estimation error, we apply the Cramér–Rao lower bound [23]. The Cramér–Rao lower bound is a fundamental lower bound on the variance of any unbiased estimator. Its calculation in general requires the Fisher information matrix F with components

(26) $$\begin{array}{cc} F_{k,\ell } & =E\left\{-\frac{\partial^{2}}{\partial a_{k}\;\partial a_{\ell }}p\left(\hat{H}\;|\;a\right)\right\}=E\left\{\frac{\partial }{\partial a_{k}}p\left(\hat{H}\;|\;a\right)\;\frac{\partial }{\partial a_{\ell }}p\left(\hat{H}\;|\;a\right)\right\}, \end{array}$$

where $p\left(\hat{H}\;|\;a\right)$ denotes the likelihood function with respect to the noisy magnetic field strength observations $\hat{H}$, and $E\left\{\;\cdot \;\right\}$ defines the expectation operator with respect to that likelihood function. Finally, the variance

(27) $$\mathrm{VAR}\left\{\hat{a_{i}}\right\}\geq {\left[F^{-1}\right]}_{i,i},$$

of an estimate $\hat{a_{i}}$ with respect to the parameter vector component $a_{i}$ is lower bounded by the *i*th diagonal element of the inverse Fisher information matrix.

### 4.1. Measurement Model

As already mentioned above, the magnetic field H depends on the position x. We are going to exploit this dependency for positioning. For that, we consider noisy measurements

(28) $$\hat{H}=H\left(x\right)+\epsilon$$

of the magnetic field strength, in particular its components. We assume that these measurements consist of the magnetic field strength $H\left(x\right)$ itself, which is disturbed by additive white Gaussian noise $\epsilon ={\left(\epsilon_{x},\epsilon_{y},\epsilon_{z}\right)}^{T}$ with zero mean and covariance

(29) $$\Sigma =E\left\{\epsilon \;\epsilon^{T}\right\}.$$

In this case, the likelihood function

(30) $$p\left(\hat{H}\;|\;x\right)=\frac{\exp\left(-\frac{1}{2}{\left(\hat{H}-H\left(x\right)\right)}^{T}\Sigma^{-1}\left(\hat{H}-H\left(x\right)\right)\right)}{\sqrt{{\left(2\pi \right)}^{n}\det\left(\Sigma \right)}}$$

is a conditional Gaussian probability density function, where *n* is the length of the error vector ϵ.

### 4.2. Fisher Information and the Cramér–Rao Lower Bound

With the spatial dependency of the magnetic field and the additive white Gaussian noise measurement model we have introduced in (28), the Fisher information matrix is calculated as [23]

(31) $$F=J{\left(x\right)}^{T}\;\Sigma^{-1}\;J\left(x\right).$$

In the case of independent and identically distributed Gaussian noise, meaning $\Sigma =\sigma^{2}I$, (31) simplifies to

(32) $$F=\frac{1}{\sigma^{2}}\;J{\left(x\right)}^{T}\;J\left(x\right).$$

The Jacobian matrix

(33) $$J\left(x\right)=\nabla H\left(x\right)$$

contains the gradients of the magnetic field strength components with respect to the spatial parameters, which we wish to estimate. By applying the gradient operator for spherical coordinates to (17), we obtain the Jacobian matrix

(34) $$\begin{array}{cc} J_{\mathrm{sph}}\left(x\right) & =\left(\frac{\partial }{\partial r},\frac{1}{r}\frac{\partial }{\partial \theta },\frac{1}{r\;\sin(\theta )}\frac{\partial }{\partial \varphi }\right)\;H\left(x\right)\\ \; & =\frac{3}{4}\;\frac{H_{\infty }}{r_{\mathrm{sph}}}\;\frac{\mu_{r}-1}{\mu_{r}+2}\;{\left(\frac{r_{\mathrm{sph}}}{r}\right)}^{4}\left(\begin{array}{ccc} -6\;\sin(2\;\theta )\;\cos(\varphi ) & 4\;\cos(2\;\theta )\;\cos(\varphi ) & -4\;\cos(\theta )\;\sin(\varphi )\\ -6\;\sin(2\;\theta )\;\sin(\varphi ) & 4\;\cos(2\;\theta )\;\sin(\varphi ) & 4\;\cos(\theta )\;\cos(\varphi )\\ -6\;\cos(2\;\theta )-1 & -4\;\sin(2\;\theta ) & 0 \end{array}\right) \end{array}$$

for a sphere in a homogeneous magnetic field, as introduced in Section 3.1.

For the cylinder case, we have calculated the magnetic field strength in Section 3.2. We observe that there is neither a dependency of the magnetic field strength on the height coordinate *z* nor a *z* component of the magnetic field strength itself. For this reason, position estimation in the *z* direction is impossible. So if we neglect the *z* dimension, we consider the remaining two-dimensional positioning problem in the *x*–*y* plane. By applying the gradient operator for the remaining polar coordinates (r,φ) to (25), we obtain the Jacobian matrix:

(35) $$\begin{array}{cc} J_{\mathrm{cyl}}\left(x\right) & =\left(\frac{\partial }{\partial r},\frac{1}{r}\frac{\partial }{\partial \varphi }\right)\;H\left(x\right)\\ \; & =2\;\frac{H_{\infty }}{r_{\mathrm{cyl}}}\;\frac{\mu_{r}-1}{\mu_{r}+1}\;{\left(\frac{r_{\mathrm{cyl}}}{r}\right)}^{3}\;\left(\begin{array}{cc} -\cos(2\;\varphi ) & -\sin(2\;\varphi )\\ -\sin(2\;\varphi ) & \cos(2\;\varphi ) \end{array}\right). \end{array}$$

With (32), the resulting Fisher information matrices are

(36) $$\begin{array}{cc} F_{\mathrm{sph}}= & \frac{9}{16\;r_{\mathrm{sph}}^{2}}\;\frac{H_{\infty }^{2}}{\sigma^{2}}\;{\left(\frac{\mu_{r}-1}{\mu_{r}+2}\right)}^{2}\;{\left(\frac{r_{\mathrm{sph}}}{r}\right)}^{8}\left(\begin{array}{ccc} 12\;\cos(2\;\theta )+37 & 4\;\sin(2\;\theta ) & 0\\ 4\;\sin(2\;\theta ) & 16 & 0\\ 0 & 0 & 16\;\cos^{2}\left(\theta \right) \end{array}\right) \end{array}$$

for the sphere and

(37) $$F_{\mathrm{cyl}}=\frac{4}{r_{\mathrm{cyl}}^{2}}\;\frac{H_{\infty }^{2}}{\sigma^{2}}\;{\left(\frac{\mu_{r}-1}{\mu_{r}+1}\right)}^{2}\;{\left(\frac{r_{\mathrm{cyl}}}{r}\right)}^{6}\;I_{2\times 2}$$

for the cylinder case with an identity matrix $I_{2\times 2}$ of dimensions 2×2. Inverting these Fisher information matrices results in the Cramér–Rao lower bound matrices

(38) $$\begin{array}{cc} C_{\mathrm{sph}}=F_{\mathrm{sph}}^{-1}= & \overset{\mathrm{location}\;\mathrm{independent}}{\overset{︷}{\frac{38}{147}\;r_{\mathrm{sph}}^{2}\;\overset{\begin{array}{c} \mathrm{magnetic}\\ \mathrm{sensor}\\ \mathrm{properties} \end{array}}{\overset{︷}{{\left(\frac{H_{\infty }^{2}}{\sigma^{2}}\right)}^{-1}}}\;\overset{\begin{array}{c} \mathrm{material}\;\mathrm{pro}-\\ \mathrm{perties},\;\sigma_{\mathrm{mat}}^{2} \end{array}}{\overset{︷}{{\left(\frac{\mu_{r}+2}{\mu_{r}-1}\right)}^{2}}}}}\times \\ & \times \underset{\mathrm{location}\;\mathrm{dependent}}{\underset{︸}{\underset{\begin{array}{c} \mathrm{radial}\;\mathrm{distance}\\ \mathrm{dependency},\;\sigma_{\mathrm{rad}}^{2} \end{array}}{\underset{︸}{{\left(\frac{r}{r_{\mathrm{sph}}}\right)}^{8}}}\;\underset{\mathrm{polar}\;\mathrm{angle}\;\mathrm{dependency}}{\underset{︸}{\left(\begin{array}{ccc} \underset{=\sigma_{r}^{2}}{\underset{︸}{\frac{392}{57\;{\left(\cos\left(2\;\theta \right)+6\right)}^{2}}}} & -\frac{98\;\sin(2\;\theta )}{57\;{\left(\cos\left(2\;\theta \right)+6\right)}^{2}} & 0\\ -\frac{98\;\sin(2\;\theta )}{57\;{\left(\cos\left(2\;\theta \right)+6\right)}^{2}} & \underset{=\sigma_{\theta }^{2}}{\underset{︸}{\frac{49\;(12\;\cos(2\;\theta )+37)}{114\;{\left(\cos\left(2\;\theta \right)+6\right)}^{2}}}} & 0\\ 0 & 0 & \underset{=\sigma_{\varphi }^{2}}{\underset{︸}{\frac{49}{114\;\cos^{2}\left(\theta \right)}}} \end{array}\right)}}}} \end{array}$$

for the sphere and

(39) $$C_{\mathrm{cyl}}=F_{\mathrm{cyl}}^{-1}=\overset{\mathrm{location}\;\mathrm{independent}}{\overset{︷}{\frac{1}{4}\;r_{\mathrm{cyl}}^{2}\;\underset{\begin{array}{c} \mathrm{magnetic}\\ \mathrm{sensor}\\ \mathrm{properties} \end{array}}{\underset{︸}{{\left(\frac{H_{\infty }^{2}}{\sigma^{2}}\right)}^{-1}}}\;\underset{\begin{array}{c} \mathrm{material}\;\mathrm{pro}-\\ \mathrm{perties},\;\sigma_{\mathrm{mat}}^{2} \end{array}}{\underset{︸}{{\left(\frac{\mu_{r}+1}{\mu_{r}-1}\right)}^{2}}}}}\;\underset{\begin{array}{c} \mathrm{radial}\;\mathrm{distance}\\ \mathrm{dependency},\;\sigma_{\mathrm{rad}}^{2} \end{array}}{\underset{︸}{{\left(\frac{r}{r_{\mathrm{cyl}}}\right)}^{6}\;}}I_{2\times 2}$$

for the cylinder.

### 4.3. Discussion of Results

Subsequently, we discuss the Cramér–Rao lower bound results of (38) and (39). The diagonal elements of the Cramér–Rao lower bound matrices provide the lower bounds for the variances of the parameter estimates. In our case, these parameter estimates are the position of the magnetic sensor in the particular coordinate system we have used. The corresponding variances describe the uncertainty along the unit vectors of the coordinate system. For the spherical coordinates, which we have used in (38), we obtain the variances in the radial ($e_{r}$), polar ($e_{\theta }$), and azimuthal ($e_{\varphi }$) direction. In the case of the cylinder coordinates, used in (39), we obtain the variances in the radial ($e_{r}$) and azimuthal ($e_{\varphi }$) direction. Note, we omit the z coordinate for the cylinder case, since there is no dependency of the magnetic field on that coordinate. This makes an estimation of the z coordinate impossible, as already discussed in Section 4.2.

From both (38) and (39), we observe that the solutions factorize into parts that depend on the location of the sensor relative to the sphere with respect to the cylinder and parts that are location independent. We have formatted these factors to be unitless and therefore describe how the position estimation variances vary with respect to the squared radius of the sphere ($r_{\mathrm{sph}}^{2}$) or the cylinder ($r_{\mathrm{cyl}}^{2}$). Subsequently, we discuss these factors mainly in standard deviation form, i.e., the square root of these variance factors.

#### 4.3.1. Dependency on Magnetic Sensor Properties

For both the sphere and the cylinder case, the solutions depend on the factor $\frac{H_{\infty }^{2}}{\sigma^{2}}$. This factor does not depend on the sensor location and can be interpreted as a signal-to-noise power ratio for the magnetic field strength measurements, which we modeled in (28). This ratio is unitless and determined by the outer magnetic field strength and the measurement noise variance $\sigma^{2}$, which is a measure for the sensor quality. As this signal-to-noise ratio increases by 10 dB, the position estimation variances decrease by one decade. In other words, the corresponding position estimation standard deviations decrease by 1decade per 20 dB signal-to-noise ratio.

#### 4.3.2. Dependency on Material Properties

**Figure 5:**
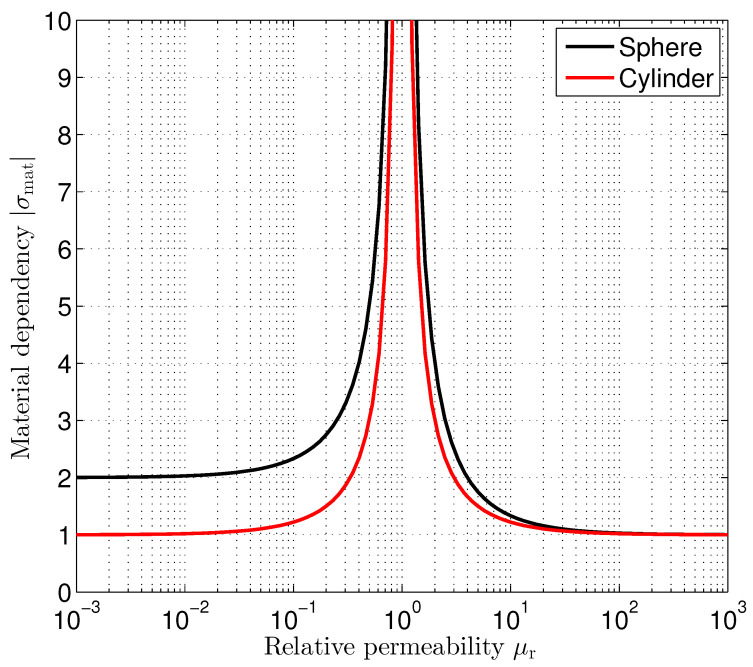
Dependency of position estimation standard deviation on the relative permeability $\mu_{r}$.

A second location-independent factor contains the permeability $\mu_{r}$ of the sphere’s respective and cylinder’s respective material relative to the outer medium. Figure 5 shows the material factor $\sigma_{\mathrm{mat}}$ for both the sphere and the cylinder. In both cases, this factor shows a pole at $\mu_{r}=1$. At $\mu_{r}=1$, the permeability of the sphere’s respective and cylinder’s respective material is identical to that of the outer medium. Consequently, there is no distortion of the outer stimulating magnetic field. It remains homogeneous and therefore location independent, which makes position estimation impossible. This means that the estimation uncertainty in the form of the estimation variances is approaching infinity. For ferromagnetic materials, $\mu_{r}\gg 1$, the material factor $\sigma_{\mathrm{mat}}$ approaches an infimum of 1. As we can observe from (38) and (39), a relative permeability of $\mu_{r}=100$ results in $\sigma_{\mathrm{mat}}=\frac{102}{99}\approx 1.03$ for the sphere and $\sigma_{\mathrm{mat}}=\frac{101}{99}\approx 1.02$ for the cylinder. So, there is only little to gain if the relative permeability is further increased. It is interesting to note that for ideal diamagnetic materials ($\mu_{r}=0$), like superconductors for example, we observe different material factor values for the sphere ($\sigma_{\mathrm{mat}}=2$) and the cylinder ($\sigma_{\mathrm{mat}}=1$).

#### 4.3.3. Dependency on Sensor Location

Previously, we have discussed those parts in the Cramér–Rao lower bounds that do not depend on the magnetic sensor location. Distortions in the magnetic field, caused by the sphere and the cylinder, are location dependent. From the Cramér–Rao lower bound theory, it is known that higher variations in the magnetic field strength provide better position estimation performance. Significant magnetic field strength variations can be observed around the magnetic structures. With increasing distance to those magnetic structures, the distortions relax, and the magnetic field becomes more and more homogeneous. Therefore, we expect higher position estimation performance around the magnetic object. Subsequently, we discuss the corresponding location-dependent factors in the Cramér–Rao lower bounds of (38) and (39).

##### Sphere

For the environment shown in Figure 1 with an outer homogeneous magnetic field in the *z* direction, we expect the resulting magnetic field to be rotationally symmetric with respect to the azimuth coordinate φ. From (13), we observe that there is neither a magnetic field component in the $e_{\varphi }$ direction nor a dependency on the azimuth coordinate φ of the remaining magnetic field components. Therefore, the position estimation performance is independent of the azimuth coordinate φ.

**Figure 6:**
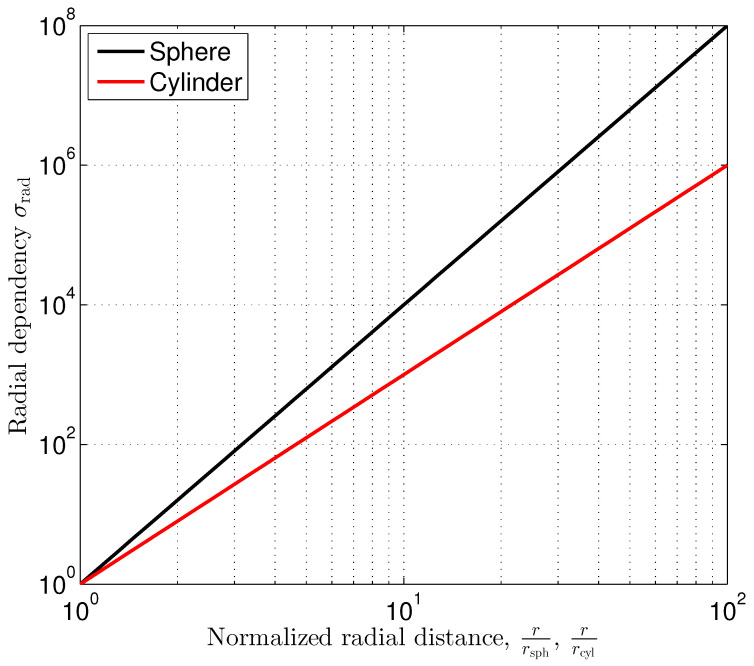
Radial dependency of position estimation standard deviation.

The radial dependency factor $\sigma_{\mathrm{rad}}$ shown in (38) increases with the 4th power of the radial distance *r* normalized to $r_{\mathrm{sph}}$, which is the radius of the sphere. So, the position estimation error quickly increases when increasing the distance to the sphere. Figure 6 shows the radial dependency $\sigma_{\mathrm{rad}}$ as a function of the normalized radial distance. The radial dependency factor affects the position estimation in all directions similarly.

**Figure 7:**
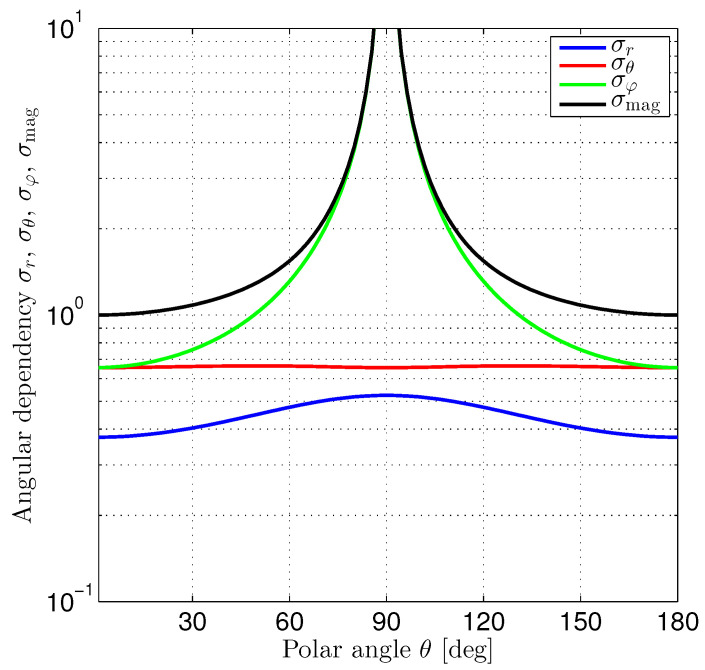
Polar angle dependency of position estimation standard deviation for a sphere in a homogeneous magnetic field.

The diagonal elements of the Cramér–Rao lower bound matrix provide the lower bounds for the variances of position estimation errors in the radial, polar, and azimuthal directions, respectively. From (38), we observe that the corresponding standard deviation factors $\sigma_{r}$, $\sigma_{\theta }$, and $\sigma_{\varphi }$ depend differently on the polar angle θ. Figure 7 shows the graphs of these factors. Additionally, we have plotted the magnitude

(40) $$\sigma_{\mathrm{mag}}=\sqrt{\sigma_{\varphi }^{2}+\sigma_{\theta }^{2}+\sigma_{r}^{2}}$$

of the position estimation error factors depending on the polar angle θ. We have normalized the polar angle-dependent diagonal elements in (38) to the magnitude’s minimum. Therefore,

(41) $$\sigma_{\mathrm{mag}}\geq 1$$

with minima ($\min\sigma_{\mathrm{mag}}=1$) occurring at $\theta =0^{\circ }$ and $\theta =180^{\circ }$.

The performance factors are limited for the radial and polar directions. In contrast, the position estimation error in the azimuthal direction shows a singularity and goes to infinity for $\theta \to 90^{\circ }$. It is interesting to note that

(42) $$\sigma_{\varphi }\;\geq \;\sigma_{\theta }\;>\;\sigma_{r},$$

with $\sigma_{\varphi }=\sigma_{\theta }$ for $\theta =0^{\circ },180^{\circ }$. According to Figure 7, position estimation in the radial direction always performs better than in the polar or azimuthal directions. The block diagonal structure of the Cramér–Rao lower bound matrix indicates that the position estimation performance in the azimuthal direction does not depend on the position estimation performances achieved in the radial and polar directions.

**Figure 8:**
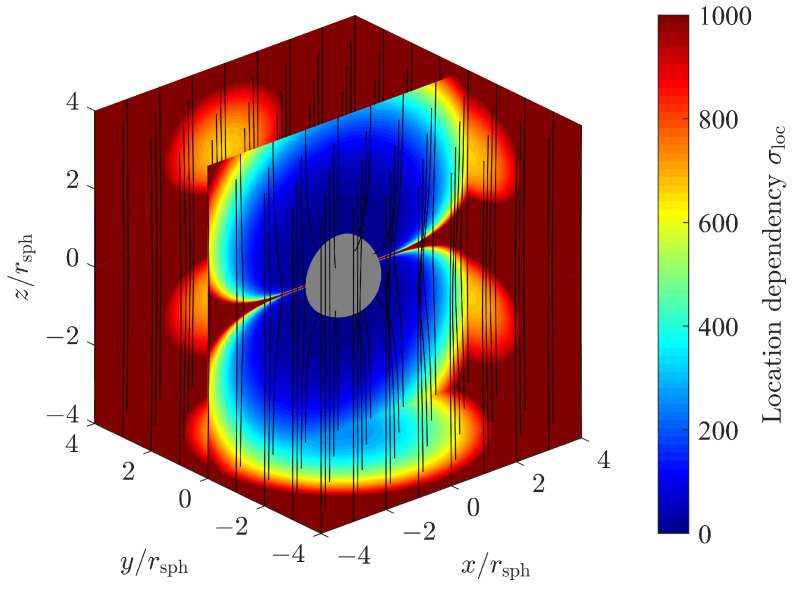
Location dependency $\sigma_{\mathrm{loc}}$ of position estimation standard deviation for a sphere in a homogeneous magnetic field.

Figure 8 shows a three-dimensional sliced color plot for the magnitude of the location-dependent position error factor, which is defined as

(43) $$\sigma_{\mathrm{loc}}=\sigma_{\mathrm{rad}}\;\sigma_{\mathrm{mag}}=\sigma_{\mathrm{rad}}\;\sqrt{\sigma_{\varphi }^{2}+\sigma_{\theta }^{2}+\sigma_{r}^{2}}.$$

Magnetic flux lines indicate the magnetic field strength and orientation. In the slice plane y=0, we observe the singularity discussed above. For the whole plane z=0, or equivalently $\theta =90^{\circ }$, the factor $\sigma_{\varphi }$ and consequently $\sigma_{\mathrm{loc}}$ go to infinity. Nevertheless, we still are able to estimate the remaining position coordinates, i.e., the radial distance *r* and the polar angle θ, with sufficient accuracy.

##### Cylinder

For the cylinder case, shown in Figure 3, the outer static magnetic field is directed along the *x* axis. The cylinder itself has infinite dilatation in the *z* direction. With this choice of the coordinate system, we obtain invariance with respect to the *z* coordinate and reduce the problem to a two-dimensional one. Consequently, position estimation in the *z* direction is not possible.

The radial dependency factor $\sigma_{\mathrm{rad}}$ shown in (39) increases with the 3th power of the radial distance *r* normalized to the cylinder radius $r_{\mathrm{cyl}}$. Compared to the sphere, which has a finite size in all the three dimensions, the impact of the inhomogeneity on the outer magnetic field, and therefore the achievable position estimation accuracy, is more significant. Figure 6 shows the corresponding graph in comparison to the sphere case.

From (21) and Figure 4, we observe that the magnetic field shows both a magnetic field component in the $e_{\varphi }$ direction and a dependency on the azimuth coordinate φ of the magnetic field components. As indicated by the identity matrix in (39) however, the position estimation error variances are equal for both the radial ($e_{r}$) and the azimuthal ($e_{\varphi }$) directions. Despite the magnetic field not being rotationally symmetric, it is interesting to see that the position estimation performance is independent of the azimuth coordinate φ in this case.

## 5. Examples

Subsequently, we consider a vertical cylindrical pillar made of ferromagnetic material as an example and assess the achievable positioning accuracy when measuring the Earth’s magnetic field in the area around that pillar.

### 5.1. Cramér–Rao Lower Bound in the Presence of Quantization Noise

We start from (39). The diagonal elements of the Cramér–Rao lower bound matrix are equal and provide the lower bounds for the variances of the position estimation in the radial ($e_{r}$) and azimuthal ($e_{\varphi }$) directions. We consider the square root of the diagonal elements and obtain

(44) $$\frac{\sigma_{r}}{r_{\mathrm{cyl}}}=\frac{\sigma_{\varphi }}{r_{\mathrm{cyl}}}=\frac{\sigma_{\mathrm{mat}}}{2}\;{\left(\frac{H_{\infty }^{2}}{\sigma_{H}^{2}}\right)}^{-\frac{1}{2}}\;{\left(\frac{r}{r_{\mathrm{cyl}}}\right)}^{3}\;$$

as the Cramér–Rao lower bound for the estimation error standard deviation in both radial and azimuthal direction normalized to the radius of the cylindrical pillar. For the position estimation standard deviation of the Cramér–Rao lower bound, we obtain

(45) $$\begin{array}{cc} \frac{\sigma_{\mathrm{pos}}}{r_{\mathrm{cyl}}}=\; & \frac{\sqrt{\sigma_{r}^{2}+\sigma_{\varphi }^{2}}}{r_{\mathrm{cyl}}}=\frac{\sqrt{\mathrm{tr}\left(C_{\mathrm{cyl}}\right)}}{r_{\mathrm{cyl}}}=\frac{\sigma_{\mathrm{mat}}}{2}\;{\left(\frac{H_{\infty }^{2}}{2\;\sigma_{H}^{2}}\right)}^{-\frac{1}{2}}\;{\left(\frac{r}{r_{\mathrm{cyl}}}\right)}^{3}\\ =\; & \frac{\sigma_{\mathrm{mat}}}{2}\;{\left(\frac{B_{\infty }^{2}}{2\;\sigma_{B}^{2}}\right)}^{-\frac{1}{2}}\;{\left(\frac{r}{r_{\mathrm{cyl}}}\right)}^{3}. \end{array}$$

In our example, the outer magnetic field $H_{\infty }$ is the Earth’s magnetic field, which we consider as sufficiently homogeneous around the pillar. We may describe the signal-to-noise ratio (SNR) terms $H_{\infty }^{2}\;/\;2\sigma_{H}^{2}=B_{\infty }^{2}\;/\;2\sigma_{B}^{2}$ in (45) either in the form of the magnetic strength *H* or the magnetic flux density *B*. Both fields are proportional, where the proportionality constant is $\mu_{0}=4\pi \;\cdot \;10^{-7}\frac{\mathrm{Vs}}{\mathrm{Am}}$ in a vacuum, which equals the permeability of air with good approximation.

The Earth’s magnetic flux density in Central Europe is approximately B=48.3μT, with a horizontal component of $B_{h}\approx 21\mu T$ and a vertical component of $B_{z}\approx 43.5\mu T$. As discussed in Section 3.2, the ferromagnetic pillar only influences the magnetic field components perpendicular to its axis. So in our case,

(46) $$B_{\infty }=\mu_{0}\;H_{\infty }=B_{h}\approx 21\mu T.$$

Next, we have a look at the measurement noise variance $\sigma_{H}^{2}$, or equivalently $\sigma_{B}^{2}$, which appears in the SNR terms in (45). As the source of measurement noise, we consider the quantization noise of the magnetic field sensor. The quantization noise variance can be calculated from the magnetic field sensor resolution as

(47) $$\sigma_{B}^{2}=\mu_{0}^{2}\;\sigma_{H}^{2}=\frac{B_{\mathrm{res}}^{2}}{12},$$

assuming that the quantization error is uniformly distributed within an interval of $\left[-\frac{B_{\mathrm{res}}}{2},+\frac{B_{\mathrm{res}}}{2}\right]$. The range and resolution of several magnetic field sensors, which are currently used in smartphones, are reported in data sheets [24,25,26,27,28]. The parameters relevant to us, together with the corresponding quantization noise variances, are summarized in Table 1.

For completeness, we have included the SNR term $H_{\infty }^{2}\;/\;2\sigma_{H}^{2}=B_{\infty }^{2}\;/\;2\sigma_{B}^{2}$ in Table 1.

**Figure 9:**
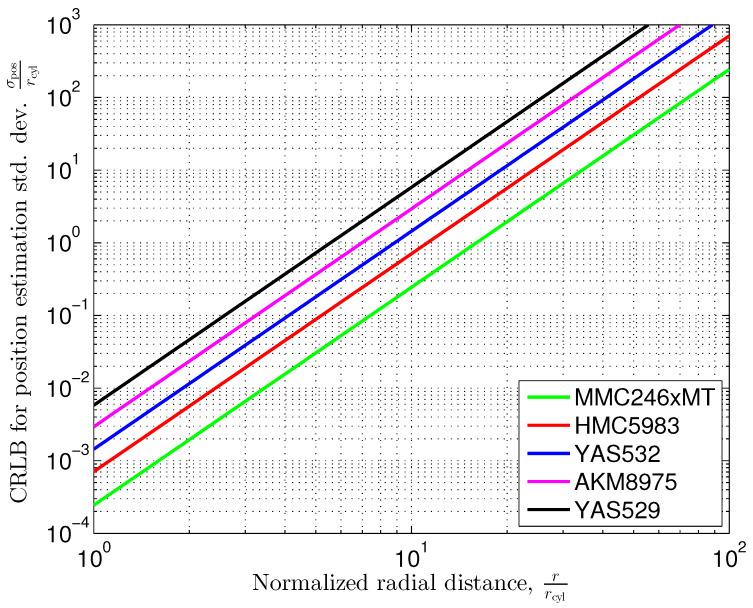
Cramér–Rao lower bound for position estimation standard deviation versus the distance of the sensor to the center of a vertical cylindrical pillar in the Earth’s magnetic field according to (45) with $\sigma_{\mathrm{mat}}=1$.

Figure 9 shows the Cramér-Rao lower bounds according to (44). The permeability of the cylinder’s material is assumed to be sufficiently large such that the material factor

(48) $$\sigma_{\mathrm{mat}}=\left|\frac{\mu_{r}+1}{\mu_{r}-1}\right|$$

becomes $\sigma_{\mathrm{mat}}\approx 1$ with good approximation.

As an example, let us consider the sensor MMC246xMT, which provides a resolution of 25 nT. This resolution results in a quantization noise variance of $52.1\;nT^{2}$ and provides a signal-to-noise ratio of $\frac{H_{\infty }^{2}}{2\;\sigma_{H}^{2}}=\frac{B_{\infty }^{2}}{2\;\sigma_{B}^{2}}=4.2\times 10^{6}=66.2$ dB with an outer horizontal Earth magnetic flux density of $B_{h}=B_{\infty }=21\mu T$. Note we express this variance in the physical unit ’Nanotesla’. We consider the physical unit ’Nanotesla’ (nT) as a whole. To avoid misinterpretations in the notation, please note that a variance of $1\;nT^{2}=1\;{\left(nT\right)}^{2}=10^{-18}\;{\left(T\right)}^{2}\;$.

To obtain a numerical example, let us assume a radius of $r_{\mathrm{cyl}}=10$ cm for the vertical pillar. From Figure 9, we observe that the Cramér–Rao lower bound for the position estimation standard deviation at a distance of $r=10\;\cdot \;r_{\mathrm{cyl}}=1\;m$ is $\sigma_{\mathrm{pos}}=0.244\;\cdot \;r_{\mathrm{cyl}}=2.44$ cm. If we further increase the distance by a factor of ten, i.e., r=10 m to the center of the cylinder, the position estimation standard deviation increases to $\sigma_{\mathrm{pos}}=24.4\;m$.

The position estimation standard deviation is proportional to the 3rd power of the (normalized) radius, thus meaning that the slope of the graphs in Figure 9 is three standard deviation decades per distance decade. For our example, position estimation at distances higher than 10 times the radius of the cylinder results in a standard deviation error, which rapidly exceeds the distance itself.

Note in Section 4 that we have derived the Cramér–Rao lower bounds for additive Gaussian noise, i.e., a Gaussian likelihood function. However, we have considered quantization and assumed a uniform likelihood function. In [29], it is shown that the Gaussian likelihood function provides the largest Cramér–Rao lower bound among all likelihood functions with equal variance. So, the Gaussian noise assumption provides a worst case.

### 5.2. Cramér–Rao Lower Bound in the Presence of Earth’s Magnetic Field Noise

We continue our example of a vertical ferromagnetic pillar. Previously, we have assumed that the Earth’s magnetic field strength, in particular its horizontal component, is known to us. The Earth’s magnetic field, however, is fluctuating. We consider these fluctuations as stochastic processes.

#### 5.2.1. Characterization of the Earth’s Magnetic Field Noise

**Figure 10:**
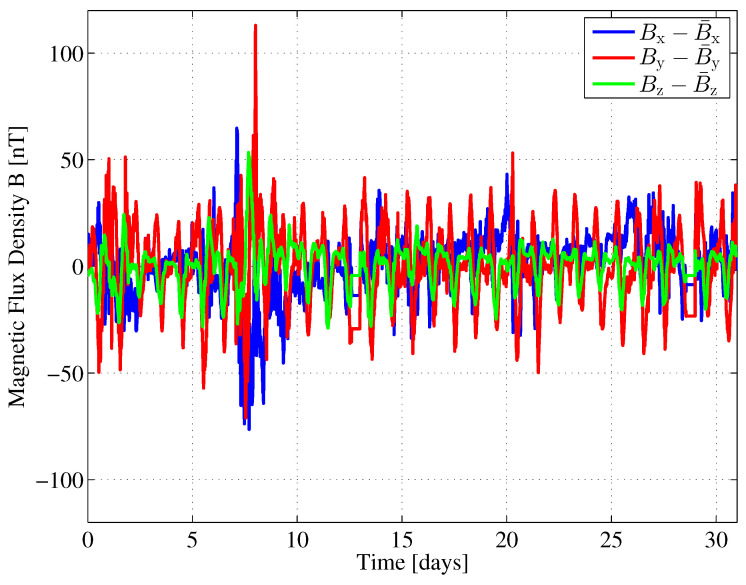
Earth’s magnetic field fluctuation in Fuerstenfeldbruck, Germany during May 2016.

These stochastic processes are the sources of measurement noise in further investigations of our example. Figure 10 shows the fluctuations

(49) $$\Delta B_{\infty }=B_{\infty }-{\bar{B}}_{\infty },$$

i.e., the differences with respect to the corresponding mean values, for the three components $B_{x}$, $B_{y}$, and $B_{z}$ of the Earth’s magnetic flux density in a Cartesian coordinate system.

**Figure 11:**
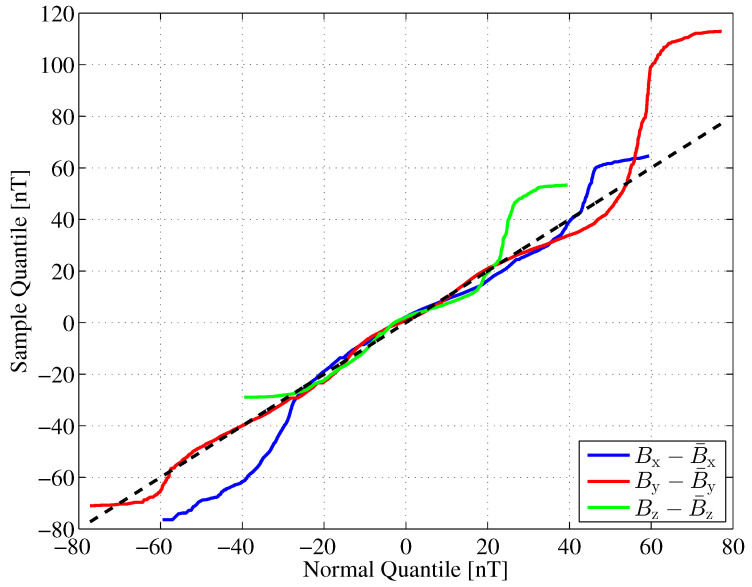
Quantile–quantile plots for the Earth magnetic field fluctuation in Fuerstenfeldbruck, Germany during May 2016.

The horizontal components $B_{x}$ and $B_{y}$ point toward the north and east, respectively. The vertical component $B_{z}$ points toward the Earth’s center. The data was obtained from an observatory in Fuerstenfeldbruck, Germany, which is part of the INTERMAGNET network [30]. The data was collected in May 2016, with a sampling interval of 1min. Out of that data, we obtain the mean vector

(50) $${\bar{B}}_{\infty }=\left(\begin{array}{c} {\bar{B}}_{x}\\ {\bar{B}}_{y} \end{array}\right)=\left(\begin{array}{c} 20968\\ 1053 \end{array}\right)\;nT$$

and covariance matrix

(51) $$\Sigma_{\infty }=E\left\{\Delta B_{\infty }\;\Delta B_{\infty }^{T}\right\}=\left(\begin{array}{cc} 212.32 & -3.0913\\ -3.0913 & 358.04 \end{array}\right)\;nT^{2}$$

of the horizontal components of the Earth’s magnetic flux density for our further investigations. The variances of the fluctuations in the *x* and *y* directions are significantly different and show only little correlation. Another statistical property of the Earth’s magnetic flux density that we are interested in is the shape of its probability density function (PDF). We compare the PDF of the Earth’s magnetic flux density fluctuations with a corresponding Gaussian PDF with equal variance. For this comparison, we draw quantile–quantile (Q-Q) plots [31], as shown in Figure 11.

If the sample data are Gaussian distributed, the Q-Q plot varies randomly around the main diagonal, which we have plotted as a dashed line. In the interval [−20,20]nT, the quantiles of the samples are quite close to those of the corresponding Gaussian distribution, thus indicating a kind of ’bell’ shape. Deviations indicate increased tails (*x* component) or skewness (*z* component) for instance.

We would like to emphasize that the analysis of the mean and variance of the Earth’s magnetic flux density is based on an exemplary one-month data sample. Depending on the geomagnetic activity and geographical location, higher fluctuations may occur than in this example. Such higher fluctuations mean higher variance values, which are regarded as noise variance in our Cramér–Rao lower bound analysis.

To summarize, the PDFs of the Earth’s magnetic flux density fluctuations are approximately, but not perfectly, Gaussian-shaped. However, as mentioned earlier, the Gaussian assumption provides a worst case assumption for calculating the Fisher information or, correspondingly, the Cramér–Rao lower bound.

#### 5.2.2. Cramér–Rao Lower Bound

For the derivation of the magnetic strength field in Section 3.2, we have assumed an outer magnetic field in the *x* direction. The Earth’s outer magnetic field shows components in both the *x* and *y* directions. With an appropriate rotation of the coordinate system, we can generalize the result in (25) and obtain

(52) $$B_{\mathrm{out}}=\left(I_{2\times 2}+\frac{1}{\sigma_{\mathrm{mat}}}\;{\left(\frac{r_{\mathrm{cyl}}}{r}\right)}^{2}\Phi \left(2\;\varphi \right)\right)\;B_{\infty }$$

with

(53) $$\Phi \left(x\right)=\left(\begin{array}{cc} \cos(x) & \sin(x)\\ \sin(x) & -\cos(x) \end{array}\right)$$

and $\sigma_{\mathrm{mat}}$, as defined in (39) and (48). Here, we describe the magnetic field in the form of the magnetic flux density $B_{\mathrm{out}}=\mu_{0}\;H_{\mathrm{out}}$ instead of the magnetic strength $H_{\mathrm{out}}$. Both fields are proportional with the proportionality constant $\mu_{0}$.

As a next step, we analyze how fluctuations of the magnetic field $B_{\infty }$ transfer into magnetic field fluctuations in the presence of a ferromagnetic pillar. According to (49), we set $B_{\infty }={\bar{B}}_{\infty }+\Delta B_{\infty }$, insert this into (52), and obtain

(54) $${\hat{B}}_{\mathrm{out}}=\left(I_{2\times 2}+\frac{1}{\sigma_{\mathrm{mat}}}\;{\left(\frac{r_{\mathrm{cyl}}}{r}\right)}^{2}\Phi \left(2\;\varphi \right)\right)\;\left({\bar{B}}_{\infty }+\Delta B_{\infty }\right).$$

as noisy magnetic flux density measurements. We identify

(55) $${\bar{B}}_{\mathrm{out}}=\left(I_{2\times 2}+\frac{1}{\sigma_{\mathrm{mat}}}\;{\left(\frac{r_{\mathrm{cyl}}}{r}\right)}^{2}\Phi \left(2\;\varphi \right)\right)\;{\bar{B}}_{\infty }$$

as the mean magnetic flux density and

(56) $$\epsilon =\left(I_{2\times 2}+\frac{1}{\sigma_{\mathrm{mat}}}\;{\left(\frac{r_{\mathrm{cyl}}}{r}\right)}^{2}\Phi \left(2\;\varphi \right)\right)\;\Delta B_{\infty }$$

as the noise part. With (56) and (51), we can calculate the noise covariance matrix as

(57) $$\begin{array}{cc} \Sigma & =E\left\{\epsilon \;\epsilon^{T}\right\}\\ \; & =\left(I_{2\times 2}+\frac{1}{\sigma_{\mathrm{mat}}}{\left(\frac{r_{\mathrm{cyl}}}{r}\right)}^{2}\Phi \right)\Sigma_{\infty }{\left(I_{2\times 2}+\frac{1}{\sigma_{\mathrm{mat}}}{\left(\frac{r_{\mathrm{cyl}}}{r}\right)}^{2}\Phi \right)}^{T}. \end{array}$$

For notational convenience, we have omitted the argument of matrix Φ. The Jacobian matrix

(58) $$\begin{array}{cc} J=\nabla {\bar{B}}_{\mathrm{out}} & =\left(\frac{\partial }{\partial r},\frac{1}{r}\frac{\partial }{\partial \varphi }\right){\bar{B}}_{\mathrm{out}}\\ & =\frac{2}{r_{\mathrm{cyl}}\;\sigma_{\mathrm{mat}}}\;{\left(\frac{r_{\mathrm{cyl}}}{r}\right)}^{3}\left[\begin{array}{cc} -\Phi \;{\bar{B}}_{\infty }\;,\; & \Phi^{\prime }\;{\bar{B}}_{\infty } \end{array}\right] \end{array}$$

of the mean magnetic flux density ${\bar{B}}_{\mathrm{out}}$ is composed of two column vectors corresponding to the derivatives of ${\bar{B}}_{\mathrm{out}}$ with respect to the radial and azimuthal directions. The derivative of matrix Φ is calculated as

(59) $$\Phi^{\prime }=\Phi^{\prime }\left(x\right)=\frac{d}{dx}\Phi \left(x\right)=\left(\begin{array}{cc} -\sin(x) & \cos(x)\\ \cos(x) & \sin(x) \end{array}\right).$$

with (57) and (58), we can calculate the Fisher information matrix $F=J^{T}\;\Sigma^{-1}\;J$ according to (31). We obtain the Cramér–Rao lower bound matrix as

(60) $$\begin{array}{cc} C\; & =F^{-1}={\left(J^{T}\;\Sigma^{-1}\;J\right)}^{-1}=J^{-1}\;\Sigma \;{J^{T}}^{-1}\\ \; & =\frac{1}{{∥{\bar{B}}_{\infty }∥}^{2}}\;{\left(\frac{r_{\mathrm{cyl}}\;\sigma_{\mathrm{mat}}}{2}\right)}^{2}\;{\left(\frac{r}{r_{\mathrm{cyl}}}\right)}^{6}\;J^{-1}\;\Sigma \;J\\ \; & ={\left(\frac{r_{\mathrm{cyl}}\;\sigma_{\mathrm{mat}}}{2\;{∥{\bar{B}}_{\infty }∥}^{2}}\right)}^{2}{\left(\frac{r}{r_{\mathrm{cyl}}}\right)}^{6}\left(\begin{array}{cc} {\bar{B}}_{\infty }^{T}\Phi \Sigma \Phi {\bar{B}}_{\infty } & -{\bar{B}}_{\infty }^{T}\Phi \Sigma \Phi^{\prime }{\bar{B}}_{\infty }\\ -{\bar{B}}_{\infty }^{T}\Phi^{\prime }\Sigma \Phi {\bar{B}}_{\infty } & {\bar{B}}_{\infty }^{T}\Phi^{\prime }\Sigma \Phi^{\prime }{\bar{B}}_{\infty } \end{array}\right) \end{array}$$

with ${∥{\bar{B}}_{\infty }∥}^{2}={\bar{B}}_{x}^{2}+{\bar{B}}_{y}^{2}$ as the squared magnitude of the mean magnetic flux density ${\bar{B}}_{\infty }$. The identities for the 2×2 matrices $J^{-1}$ and ${J^{T}}^{-1}$ are derived in Appendix A.

#### 5.2.3. Discussion of Results

**Figure 12:**
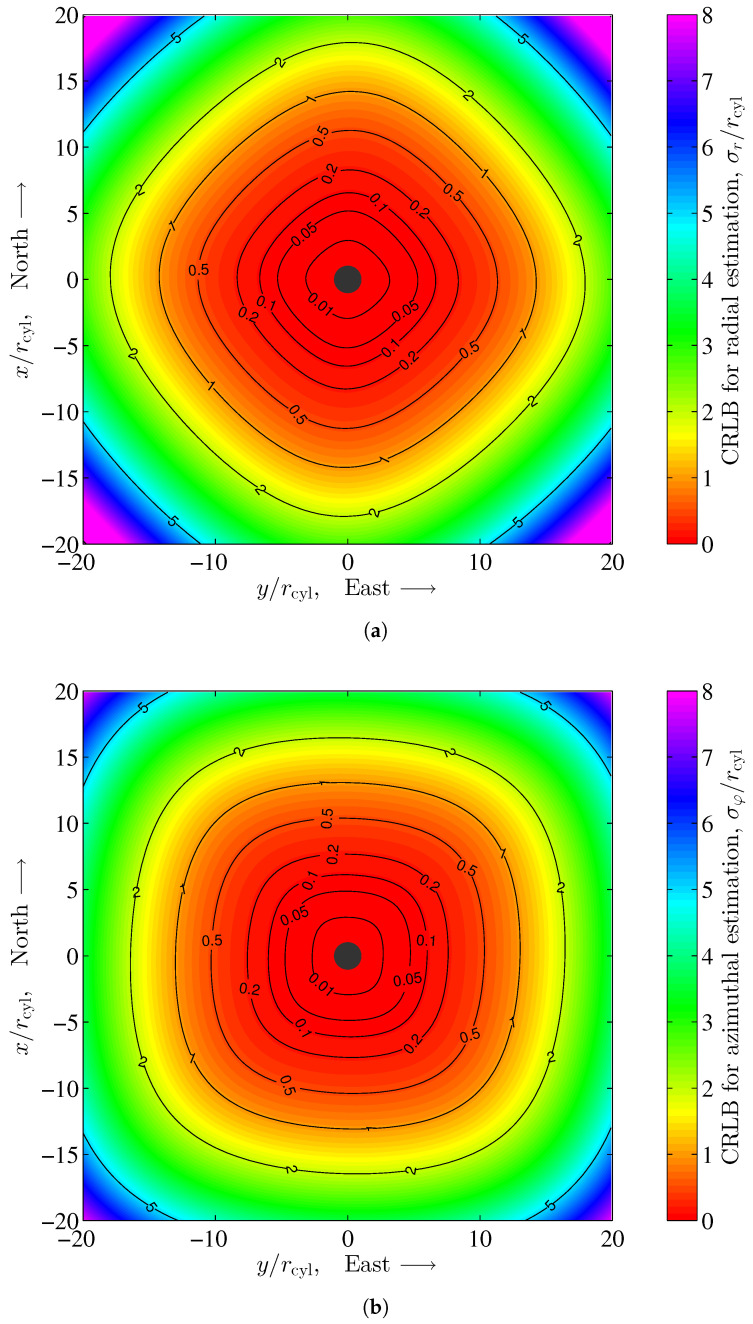
Cramér–Rao lower bound for position estimation standard deviation of the radial and azimuthal component for a vertical cylindrical pillar in the Earth’s magnetic field according to (61) and (62), with $\sigma_{\mathrm{mat}}=1$. (**a**) Radial estimation; (**b**) Azimuthal estimation.

The square root of the main diagonal elements of matrix C in (60) provide the Cramér–Rao lower bound for the standard deviation

(61) $$\frac{\sigma_{r}}{r_{\mathrm{cyl}}}=\frac{\sqrt{C_{1,1}}}{r_{\mathrm{cyl}}}=\frac{\sigma_{\mathrm{mat}}}{2\;{∥{\bar{B}}_{\infty }∥}^{2}}\;{\left(\frac{r}{r_{\mathrm{cyl}}}\right)}^{3}\;\sqrt{{\bar{B}}_{\infty }^{T}\Phi \Sigma \Phi {\bar{B}}_{\infty }}$$

for the radial estimation error and

(62) $$\frac{\sigma_{\varphi }}{r_{\mathrm{cyl}}}=\frac{\sqrt{C_{2,2}}}{r_{\mathrm{cyl}}}=\frac{\sigma_{\mathrm{mat}}}{2\;{∥{\bar{B}}_{\infty }∥}^{2}}\;{\left(\frac{r}{r_{\mathrm{cyl}}}\right)}^{3}\;\sqrt{{\bar{B}}_{\infty }^{T}\Phi^{\prime }\Sigma \Phi^{\prime }{\bar{B}}_{\infty }}$$

for the azimuthal estimation error. For a more general description, the Cramér–Rao lower bounds in (61) and (62) are normalized to the radius $r_{\mathrm{cyl}}$ of the cylinder. In Section 4, we have assumed a noise covariance of form $\Sigma =\sigma^{2}I$. With this assumption, the noise variance is constant, i.e., independent of the location of the sensor, and its components in both the *x* and *y* directions are equal. However, the noise covariance, shown in (57) for our example, depends on the location of the sensor through variable *r* and the argument of matrix Φ, which in general show different main diagonal elements through (51). Compared to the evaluations in Section 4, the Cramér–Rao lower bounds for both the radial and azimuthal estimations are dependent on the azimuth φ as well. Figure 12 shows the Cramér–Rao lower bounds for the position estimation standard deviations of the radial and azimuthal components for a vertical cylindrical pillar in the Earth’s magnetic field according to (61) and (62) for our example. The dependency on the azimuth φ is clearly visible, since the contour lines are not circularly shaped.

**Figure 13:**
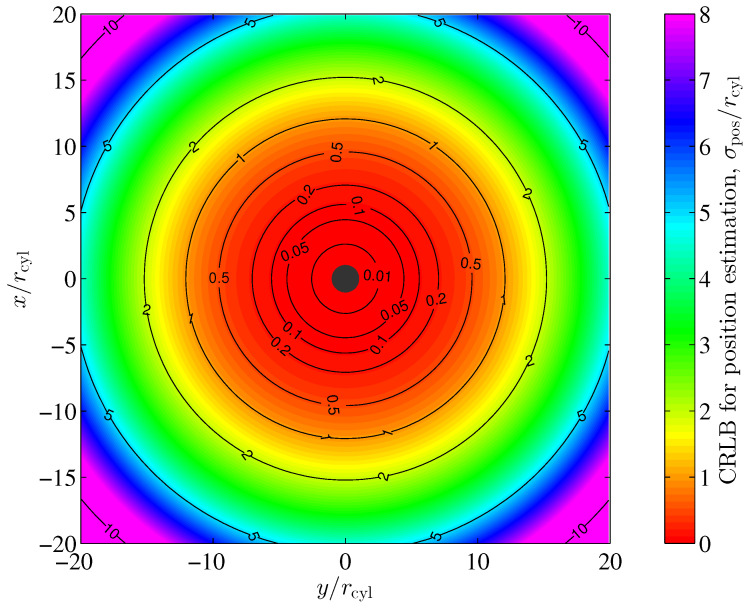
Cramér–Rao lower bound for position estimation standard deviation for a vertical cylindrical pillar in the Earth’s magnetic field according to (63) with $\sigma_{\mathrm{mat}}=1$.

For the calculation of the position estimation standard deviation of the Cramér–Rao lower bound

(63) $$\begin{array}{cc} \frac{\sigma_{\mathrm{pos}}}{r_{\mathrm{cyl}}} & =\frac{\sqrt{\sigma_{r}^{2}+\sigma_{\varphi }^{2}}}{r_{\mathrm{cyl}}}=\frac{\sqrt{\mathrm{tr}\left(C\right)}}{r_{\mathrm{cyl}}}=\frac{\sigma_{\mathrm{mat}}}{2}\;{\left(\frac{r}{r_{\mathrm{cyl}}}\right)}^{3}\sqrt{\frac{\mathrm{tr}\left(\Sigma \right)}{{∥{\bar{B}}_{\infty }∥}^{2}}}\\ \; & =\frac{\sigma_{\mathrm{mat}}}{2}\;{\left(\frac{r}{r_{\mathrm{cyl}}}\right)}^{3}\sqrt{\frac{\mathrm{tr}\left(\left(I_{2\times 2}+\frac{1}{\sigma_{\mathrm{mat}}}{\left(\frac{r_{\mathrm{cyl}}}{r}\right)}^{2}\Phi \right)\Sigma_{\infty }{\left(I_{2\times 2}+\frac{1}{\sigma_{\mathrm{mat}}}{\left(\frac{r_{\mathrm{cyl}}}{r}\right)}^{2}\Phi \right)}^{T}\right)}{{∥{\bar{B}}_{\infty }∥}^{2}}} \end{array}$$

we have started with the 2nd line in (60) and used the identity $\mathrm{tr}\left(J^{-1}\Sigma J\right)=\mathrm{tr}\left(\Sigma JJ^{-1}\right)=\mathrm{tr}\left(\Sigma \right)$ for the trace operator. As in the case of the radial and azimuthal estimations, the position estimation of the Cramér–Rao lower bound according to (63) depends on the azimuth φ through matrix Φ. However, the influence of matrix Φ for the calculation of the Earth’s magnetic field noise covariance matrix Σ rapidly decreases with the 2nd power of radius *r*, i.e., the distance of the sensor to the center of the cylinder. Figure 13 shows the position estimation of the Cramér–Rao lower bound according to (63). A significant dependency on the azimuth φ is not visible.

For sufficiently large distances *r*, the position estimation of the Cramér–Rao lower bound approaches the asymptotic value

(64) $$\lim_{r\to \infty }\frac{\sigma_{\mathrm{pos}}}{r_{\mathrm{cyl}}}=\frac{\sigma_{\mathrm{mat}}}{2}\;{\left(\frac{{∥{\bar{B}}_{\infty }∥}^{2}}{\mathrm{tr}\left(\Sigma_{\infty }\right)}\right)}^{-\frac{1}{2}}{\left(\frac{r}{r_{\mathrm{cyl}}}\right)}^{3},$$

which is similar to (45) in its structure. In particular, we observe that the asymptotic position estimation of the Cramér–Rao lower bound is independent of the azimuth φ. For our example, the SNR term in (64) becomes

(65) $$\frac{{∥{\bar{B}}_{\infty }∥}^{2}}{\mathrm{tr}\left(\Sigma_{\infty }\right)}=\frac{\left(20968^{2}+1053^{2}\right)\;{nT}^{2}}{\left(212.32+358.04\right)\;{nT}^{2}}\;=7.7\;\cdot \;10^{5}.$$

Note that the fluctuations in the Earth’s magnetic flux density are accounted for by the covariance matrix **Σ**_∞_. Higher fluctuations lead to a higher trace, tr(**Σ**_∞_), of that covariance matrix and consequently to a lower SNR and an increased Cramér–Rao lower bound.

### 5.3. Cramér–Rao Lower Bound in the Presence of Quantization and Magnetic Field Noise

**Figure 14:**
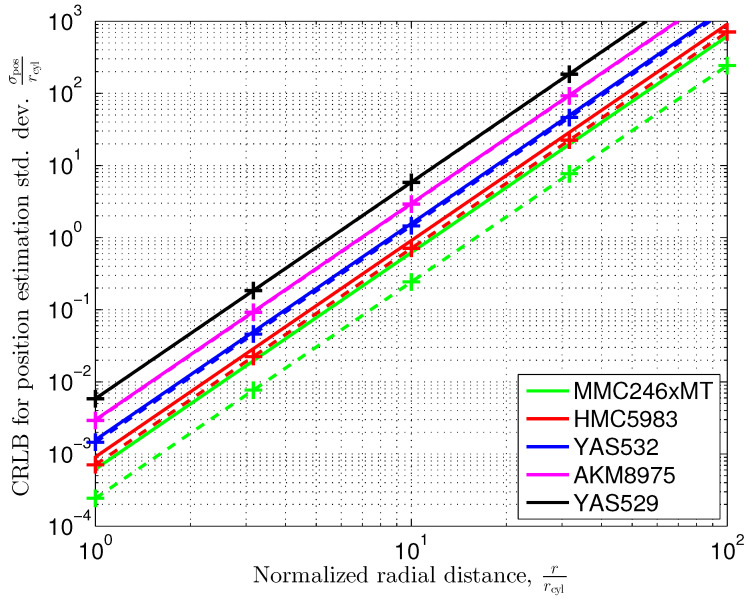
Cramér–Rao lower bound for position estimation standard deviation for a vertical cylindrical pillar in the Earth’s magnetic field according to (64), with (66) and $\sigma_{\mathrm{mat}}=1$. Solid lines represent results for considering the Earth’s magnetic field noise and the sensors’ quantization noise. Dashed lines with markers (’+’) repeat results from Figure 9, where only quantization noise has been considered.

For the consideration of both the quantization noise and the Earth’s magnetic field noise, we calculate a general noise covariance

(66) $$\Sigma =\left(I_{2\times 2}+\frac{1}{\sigma_{\mathrm{mat}}}{\left(\frac{r_{\mathrm{cyl}}}{r}\right)}^{2}\Phi \right)\;\Sigma_{\infty }\;{\left(I_{2\times 2}+\frac{1}{\sigma_{\mathrm{mat}}}{\left(\frac{r_{\mathrm{cyl}}}{r}\right)}^{2}\Phi \right)}^{T}+\sigma_{B}^{2}\;I_{2\times 2}$$

which we then can insert into (61)–(63), thus replacing $\Sigma_{\infty }$. With the addition of the corresponding covariance matrices in (66), we consider the magnetic field noise and sensor quantization noise to be uncorrelated. For the asymptotic case, we have plotted the Cramér–Rao lower bound according to (64) but replaced $\Sigma_{\infty }$ with Σ in (66). The graphs are shown in Figure 14 as solid lines. For comparison, we have included the results that have been obtained for the different sensors in Section 5.1 (Figure 9), where only the quantization noise has been taken into account (dashed line with markers). When comparing the magnetic field noise variance $\mathrm{tr}\left(\Sigma_{\infty }\right)=570.36\;nT^{2}$ with the sensor quantization noise $\mathrm{tr}\left(\sigma_{B}^{2}\;I_{2\times 2}\right)=2\;\sigma_{B}^{2}$, with $\sigma_{B}^{2}$ summarized in Table 1, we observe that for the sensors AKM8975, YAS529, and YAS532, the sensor quantization noise is dominant. Therefore, we approximately obtain the performance derived in Section 5.1 for the sensors AKM8975, YAS529, and YAS532. This is clearly visible in Figure 14. The results when considering quantization and magnetic field noise match with the corresponding results when taking into account quantization noise only (black, magenta, and blue dashed lines with markers), and the graphs overlap. For the sensor HMC5983, the Earth’s magnetic field noise and the sensor quantization noise variances are within a similar order of magnitude. An additional performance degradation when taking into account the Earth’s magnetic field noise (red line) in addition to the sensor quantization noise can be observed in Figure 14. When applying sensors with significantly lower sensor noise, like the MMC246xMT, the Earth’s magnetic field noise becomes dominant. Therefore, we can expect the results derived in Section 5.2 to have good approximation.

In our evaluations above, we have considered sensor quantization and fluctuations in the magnetic flux density of the Earth as sources of noise. Electrical currents in the environment can also lead to impairments in magnetic field-based position determination. The resulting magnetic fields are superimposed on the Earth’s magnetic field and lead to higher magnetic field noise and therefore to higher CRLBs.

## 6. Summary and Conclusions

In this paper, we aimed at assessing the achievable positioning performance based on measuring the ambient magnetic field around elementary ferromagnetic structures, in particular a sphere and a cylinder, from a theoretical point of view. For that, we have calculated the Cramér–Rao lower bound for the position estimation around a sphere and a cylinder placed in an outer homogeneous magnetic field. For these structures, the magnetic field is analytically known.

We have seen that usual ferromagnetic materials like ordinary steel with a relative permeability of several thousands are sufficient. Using specifically designed ferromagnetic materials like permalloy, a nickel–iron magnetic alloy with $\mu_{r}\approx 10^{5}$, provides only little additional performance improvements.

The results have also shown that the position estimation error variance is proportional to the measurement noise variance. It is obvious that the sensor itself is a source of measurement noise. In this paper, we have considered the sensor quantization noise, which can differ by several orders of magnitude for different sensors. For the case that the instantaneous outer magnetic field strength is not known perfectly, we have considered outer magnetic field fluctuations as another source of measurement noise. In an example, we have observed an outer Earth magnetic field variance of $570.36\;nT^{2}$ of the horizontal component. For low resolution sensors, the outer magnetic field variance can be neglected. For high resolution sensors, the outer Earth magnetic field might be dominating. In order to take advantage of a high sensor resolution, the instantaneous Earth magnetic field strength has to be known. A potential solution is to simultaneously estimate that value with the help of a magnetic sensor array instead of a single sensor.

The influence of a magnetic structure to the ambient magnetic field relaxes with increasing distance. Therefore, the position estimation performance also decreases. For a ferromagnetic sphere in an outer homogeneous magnetic field, we have seen that the position estimation error variance increases by eight decades per distance decade. Correspondingly, the position estimation standard deviation increases by four decades per distance decade. Compared to a sphere, which has a limited size in all three dimensions, we additionally investigated a cylinder with infinite size in one dimension. For the cylinder case, the position estimation standard deviation increases by three decades per distance decade. However, position estimation is only possible in two dimensions, i.e., the plane perpendicular to the cylinder. Due to the rapidly increasing error, position estimation with sufficient accuracy is possible only in close proximity of such ferromagnetic structures. Both the position estimation standard deviation and the corresponding distance are proportional to the size of the structure, which is in our case its radius.

In this paper, we have focused on elementary structures that allow for the analytic description of the ambient magnetic field. This provided us with some general insights into the positioning error and its principle dependencies on relevant parameters like the permeability of the material. In practice, however, we face structures that are much more complex. Such structures require a numerical calculation of their ambient magnetic field and are out of the scope of this paper. Once the magnetic field has been calculated, the methods used in this paper for assessing the achievable position estimation performance, in particular the Cramér–Rao lower bound, can be applied in a similar way. Undoubtedly, it is interesting to experimentally verify the research questions raised in Section 1.2 for the considered setups of a ferromagnetic sphere or cylinder in an outer homogeneous magnetic field. As we focus on the theoretical aspects in this paper, such experimental support is the subject of further work.

## Figures and Tables

**Table 1:** Magnetic field sensors’ characteristics.

Sensor	Ref.	Range [μT]	Resolution $B_{\mathrm{res}}$ [nT]	Quant. Noise $\sigma_{B}^{2}$ [${\mathrm{nT}}^{2}$]	SNR $\frac{H_{\infty }^{2}}{2\sigma_{H}^{2}}=\frac{B_{\infty }^{2}}{2\sigma_{B}^{2}}$
MMC246xMT	[24]	±600	25	52.1	$4.2\times 10^{6}$
HMC5983	[25]	±88	73	444	$5.0\times 10^{5}$
YAS532	[26]	±1200	x,y: 150	1875	$1.2\times 10^{5}$
			z: 250	5208	
AKM8975	[27]	±1200	300	7500	$2.9\times 10^{4}$
YAS529	[28]	±300	x,y: 600	30,000	$7.4\times 10^{3}$
			z: 1200	120,000	

## Data Availability

The source of the raw data used for characterization of the Earth’s magnetic field noise is referenced within the article.

## Appendix A. Properties of the Jacobian Matrix for a Cylinder in a Homogeneous Magnetic Field

By construction, the matrices

(A1) $$\Phi =\left(\begin{array}{cc} \cos(x) & \sin(x)\\ \sin(x) & -\cos(x) \end{array}\right)\;\mathrm{and}\;\Phi^{\prime }=\left(\begin{array}{cc} -\sin(x) & \cos(x)\\ \cos(x) & \sin(x) \end{array}\right)$$

are orthogonal and symmetric, i.e., $\Phi =\Phi^{T}=\Phi^{-1}$ and $\Phi^{\prime }={\Phi^{\prime }}^{T}={\Phi^{\prime }}^{-1}$. In particular,

(A2) $$\Phi \;\Phi =I\;\mathrm{and}\;\Phi^{\prime }\;\Phi^{\prime }=I.$$

We can also verify that for an arbitrary vector a,

(A3) $$a^{T}\Phi \;\Phi^{\prime }a=0$$

holds. With the definition of matrix J in (58) we calculate

(A4) $$\begin{array}{cc} J^{T}\;J\; & ={\left(\frac{2}{r_{\mathrm{cyl}}\;\sigma_{\mathrm{mat}}}\right)}^{2}\;{\left(\frac{r_{\mathrm{cyl}}}{r}\right)}^{6}\;\left(\begin{array}{c} -{\bar{B}}_{\infty }^{T}\;\Phi \\ -{\bar{B}}_{\infty }^{T}\;\Phi^{\prime } \end{array}\right)\;\left(\begin{array}{cc} -\Phi \;{\bar{B}}_{\infty }\;,\; & \Phi^{\prime }\;{\bar{B}}_{\infty } \end{array}\right)\\ \; & ={\left(\frac{2}{r_{\mathrm{cyl}}\;\sigma_{\mathrm{mat}}}\right)}^{2}\;{\left(\frac{r_{\mathrm{cyl}}}{r}\right)}^{6}\;\left(\begin{array}{cc} {\bar{B}}_{\infty }^{T}\Phi \;\Phi {\bar{B}}_{\infty } & -{\bar{B}}_{\infty }^{T}\Phi \;\Phi^{\prime }{\bar{B}}_{\infty }\\ -{\bar{B}}_{\infty }^{T}\Phi^{\prime }\;\Phi {\bar{B}}_{\infty } & {\bar{B}}_{\infty }^{T}\Phi^{\prime }\;\Phi^{\prime }{\bar{B}}_{\infty } \end{array}\right). \end{array}$$

with the properties of the matrices Φ and $\Phi^{\prime }$ and after division of the right-hand side scalar factor we obtain

(A5) $$\underset{=J^{-1}}{\underset{︸}{\frac{1}{{∥{\bar{B}}_{\infty }∥}^{2}}\;{\left(\frac{r_{\mathrm{cyl}}\;\sigma_{\mathrm{mat}}}{2}\right)}^{2}\;{\left(\frac{r}{r_{\mathrm{cyl}}}\right)}^{6}\;J^{T}}}\;J=I.$$

From the identity for $J^{-1}$ above, we directly obtain

(A6) $$\begin{array}{cc} {J^{T}}^{-1}\; & =\frac{1}{{∥{\bar{B}}_{\infty }∥}^{2}}\;{\left(\frac{r_{\mathrm{cyl}}\;\sigma_{\mathrm{mat}}}{2}\right)}^{2}\;{\left(\frac{r}{r_{\mathrm{cyl}}}\right)}^{6}\;J=\frac{r_{\mathrm{cyl}}\;\sigma_{\mathrm{mat}}}{2\;{∥{\bar{B}}_{\infty }∥}^{2}}\;{\left(\frac{r}{r_{\mathrm{cyl}}}\right)}^{3}\;\left[\begin{array}{cc} -\Phi \;{\bar{B}}_{\infty }\;,\; & \Phi^{\prime }\;{\bar{B}}_{\infty }. \end{array}\right]. \end{array}$$

## Appendix B. A Sphere in a Homogenous Magnetic Field

We assume a sphere with radius $r_{\mathrm{sph}}$, located at the origin of a spherical coordinate system as shown in Figure 1.

### Appendix B.1. Solution of Laplace’s Equation in Spherical Coordinates

According to the method of separation of variables we assume that a solution

(A7) Ψ(r,θ,φ)=R(r)Θ(θ)Φ(φ)

for Laplace’s equation can be factorized into three terms, each depending on one coordinate variable only. With this approach, we obtain Laplace’s partial differential equation in spherical coordinates as

(A8) $$0=\Delta \Psi \left(r,\theta ,\varphi \right)=\left(\begin{array}{c} \frac{1}{r^{2}}\frac{\partial }{\partial r}r^{2}\frac{\partial }{\partial r}+\frac{1}{r^{2}\sin\left(\theta \right)}\frac{\partial }{\partial \theta }\sin\left(\theta \right)\frac{\partial }{\partial \theta }+\frac{1}{r^{2}\;\sin^{2}\left(\theta \right)}\;\frac{\partial^{2}}{\partial \varphi^{2}} \end{array}\right)\;R\left(r\right)\;\Theta \left(\theta \right)\;\Phi \left(\varphi \right).$$

#### Appendix B.1.1. Separation of the Laplace Equation

Applying the Laplace operator and multiplying by $\frac{r^{2}}{R(r)\;\Theta (\theta )\;\Phi (\varphi )}$ yields

(A9) $$0=\frac{1}{R(r)}\frac{\partial }{\partial r}r^{2}\frac{\partial }{\partial r}R\left(r\right)+\frac{1}{\sin(\theta )\Theta (\theta )}\frac{\partial }{\partial \theta }\sin\left(\theta \right)\frac{\partial }{\partial \theta }\Theta \left(\theta \right)+\frac{1}{\sin^{2}\left(\theta \right)\Phi \left(\varphi \right)}\;\frac{\partial^{2}}{\partial \varphi^{2}}\Phi \left(\varphi \right).$$

The first term depends only on *r* whereas the second and third term depends on the angular coordinates θ and φ. (A9) can only hold if both the radial and the angular component are constant. Therefore, (A9) separates into an ordinary differential equation

(A10) $$\frac{1}{R(r)}\frac{\partial }{\partial r}r^{2}\frac{\partial }{\partial r}R\left(r\right)-n\left(n+1\right)=0$$

for the radial part and into a partial differential equation

(A11) $$\frac{1}{\sin(\theta )\Theta (\theta )}\frac{\partial }{\partial \theta }\sin\left(\theta \right)\frac{\partial }{\partial \theta }\Theta \left(\theta \right)+\frac{1}{\sin^{2}\left(\theta \right)\Phi \left(\varphi \right)}\;\frac{\partial^{2}}{\partial \varphi^{2}}\Phi \left(\varphi \right)+n\left(n+1\right)=0$$

for the angular part. Finite solutions for the angular part only exist if the separation constant is an integer value of form n(n+1) [22]. Multiplying by $\sin^{2}\left(\theta \right)$, (A11) can be separated further into an azimuth part

(A12) $$\frac{1}{\Phi (\varphi )}\;\frac{\partial^{2}}{\partial \varphi^{2}}\Phi \left(\varphi \right)+m^{2}=0.$$

and a polar part

(A13) $$n\left(n+1\right)\;\sin^{2}\left(\theta \right)+\frac{\sin(\theta )}{\Theta (\theta )}\frac{\partial }{\partial \theta }\sin\left(\theta \right)\frac{\partial }{\partial \theta }\Theta \left(\theta \right)-m^{2}=0.$$

#### Appendix B.1.2. Solution of the Polar Part

Multiplying by $\frac{\Theta (\theta )}{\sin^{2}\left(\theta \right)}$ and substituting $x=\cos\left(\theta \right),\sqrt{1-x^{2}}=\sin\left(\theta \right),\partial x=-\sin\left(\theta \right)\partial \theta$ yields Legendre’s differential equation

(A14) $$\frac{\partial }{\partial x}\left(1-x^{2}\right)\frac{\partial }{\partial x}P\left(x\right)+\left(n\left(n+1\right)-\frac{m^{2}}{\left(1-x^{2}\right)}\right)P\left(x\right)=0,$$

where P(x) is obtained from Θ(θ) by substituting θ accordingly. Solutions of this differential equations are the associated Legendre polynomials

(A15) $$P_{n}^{m}\left(x\right)=\frac{{\left(1-x^{2}\right)}^{m/2}}{2^{n}\;n!}\;\frac{\partial^{m+n}}{\partial x^{m+n}}{\left(x^{2}-1\right)}^{n},\;0\leq m\leq n,$$

which are a complete set of orthogonal functions in the interval −1≤x≤+1.

#### Appendix B.1.3. Solution of the Azimuth Part

A solution to (A12) is

(A16) $$\Phi_{m}\left(\varphi \right)=A_{m}\;\cos\left(m\varphi \right)+B_{m}\;\sin\left(m\varphi \right).$$

This solution must be periodic with a period of 2π. Thus, *m* must be an integer value.

#### Appendix B.1.4. Solution of the Radial Part

A solution for the radial Equation (A10) is

(A17) $$R\left(r\right)=C_{n}\;r^{n}+D_{n}\;\frac{1}{r^{n+1}}.$$

#### Appendix B.1.5. The General Solution

For the general solution we combine (A15), (A16), (A17) and get

(A18) $$\Psi \left(r,\theta ,\varphi \right)=\sum_{n=0}^{\infty }\sum_{m=0}^{n}\left(C_{n}\;r^{n}+D_{n}\;\frac{1}{r^{n+1}}\right)\;P_{n}^{m}\left(\cos\left(\theta \right)\right)\left(A_{m}\cos\left(m\varphi \right)+B_{m}\sin\left(m\varphi \right)\right)$$

### Appendix B.2. Solution of the Boundary Value Problem

We assume a homogeneous magnetic strength vector field in z-direction with magnitude $H_{\infty }$ which we can express as

(A19) $$H_{\infty }=H_{\infty }\;e_{z}=H_{\infty }\cos\left(\theta \right)\;e_{r}-H_{\infty }\sin\left(\theta \right)\;e_{\theta }$$

in Cartesian respectively spherical coordinates, which follows from the corresponding magnetic potential field

(A20) $$\Psi_{\infty }=-H_{\infty }\;z=-H_{\infty }\;r\;\cos\left(\theta \right).$$

with this choice, the resulting magnetic potential field must be rotationally symmetric in azimuth direction. For this reason, the constant

(A21) m=0

which lets the azimuth part in (A18) vanish. We start with the magnetic potential field outside the sphere. In order to fulfill the boundary constraints (A20) we require with the remaining part of (A18)

(A22) $$\lim_{r\to \infty }\Psi_{\mathrm{out}}=\lim_{r\to \infty }\sum_{n=0}^{\infty }\left(C_{n}\;r^{n}+D_{n}\;\frac{1}{r^{n+1}}\right)\;P_{n}^{0}\left(\cos\left(\theta \right)\right)\overset{!}{=}-H_{\infty }\;r\;\cos\left(\theta \right),$$

where $C_{n}$ and $D_{n}$ are constants to be determined for the outer field. Since $P_{1}^{0}\left(x\right)=x$, we observe that

(A23) $$C_{n}=\left\{\begin{array}{cc} -H_{\infty }\; & n=1,\\ 0\; & n\neq 1. \end{array}\right.$$

The inner potential field

(A24) $$\Psi_{\mathrm{in}}=\sum_{n=0}^{\infty }\left({\tilde{C}}_{n}\;r^{n}+{\tilde{D}}_{n}\;\frac{1}{r^{n+1}}\right)\;P_{n}^{0}\left(\cos\left(\theta \right)\right)$$

requires

(A25) $${\tilde{D}}_{n}=0.$$

Otherwise, the magnetic potential diverges for r→0.

For n≠1 we have $C_{n}=0$. The boundary conditions at the sphere’s surface $\left(r=r_{\mathrm{sph}},0\leq \theta \leq \pi ,0\leq \varphi \;<2\pi \right)$ from (5) respectively (6) yield the equation system

(A26) $$\begin{array}{cccc} r_{\mathrm{sph}}^{n}\;{\tilde{C}}_{n} & - & \frac{1}{r_{\mathrm{sph}}^{n+1}}\;D_{n}\; & =0\\ \mu_{\mathrm{in}}\;n\;r_{\mathrm{sph}}^{n-1}\;{\tilde{C}}_{n} & + & \frac{\mu_{\mathrm{out}}\;\left(n+1\right)}{r_{\mathrm{sph}}^{n+2}}\;D_{n}\; & =0 \end{array}$$

This system of equation has full rank and, therefore, yields to the trivial solution ${\tilde{C}}_{n}=0$ and $D_{n}=0$ only. For n=1, however, $C_{1}=-H_{\infty }$ and we get

(A27) $$\begin{array}{ccccc} {\tilde{C}}_{1} & - & \frac{1}{r_{\mathrm{sph}}^{3}}\;D_{1}\; & =\; & -H_{\infty }\\ \frac{\mu_{\mathrm{in}}}{\mu_{\mathrm{out}}}\;{\tilde{C}}_{1} & + & \frac{2}{r_{\mathrm{sph}}^{3}}\;D_{1}\; & =\; & -H_{\infty } \end{array}$$

Solving the equations above provides

(A28) $$\begin{array}{cccc} {\tilde{C}}_{1} & =-H_{\infty }\;\frac{3}{\frac{\mu_{\mathrm{in}}}{\mu_{\mathrm{out}}}+1}\; & D_{1} & =H_{\infty }\frac{\frac{\mu_{\mathrm{in}}}{\mu_{\mathrm{out}}}+1}{\frac{\mu_{\mathrm{in}}}{\mu_{\mathrm{out}}}+1}\;r_{\mathrm{sph}}^{3} \end{array}$$

and leads to the scalar magnetic potential fields

(A29) $$\begin{array}{cc} \Psi_{\mathrm{in}}\; & =-H_{\infty }\;\;\frac{3}{\frac{\mu_{\mathrm{in}}}{\mu_{\mathrm{out}}}+2}\;r\;\cos\left(\theta \right) \end{array}$$

(A30) $$\begin{array}{cc} \Psi_{\mathrm{out}} & =H_{\infty }\left(\frac{\frac{\mu_{\mathrm{in}}}{\mu_{\mathrm{out}}}-1}{\frac{\mu_{\mathrm{in}}}{\mu_{\mathrm{out}}}+2}{\left(\frac{r_{\mathrm{sph}}}{r}\right)}^{3}-1\right)\;r\;\cos\left(\theta \right) \end{array}$$

inside and outside the sphere.

## Appendix C. A Cylinder in a Homogenous Magnetic Field

We assume a cylinder of infinite length with radius $r_{\mathrm{cyl}}$, located along the z-axis of a cylindrical coordinate system as shown in Figure 3.

### Appendix C.1. Solution of Laplace’s Equation in Cylinder Coordinates

According to the method of separation of variables we assume that a solution

(A31) Ψ(r,φ,z)=R(r)Φ(φ)Z(z)

for Laplace’s equation can be factorized into three terms, each depending on one coordinate variable only. With this approach, we obtain Laplace’s partial differential equation in cylinder coordinates as

(A32) $$0=\Delta \Psi \left(r,\varphi ,z\right)=\left(\begin{array}{c} \frac{1}{r}\frac{\partial }{\partial r}r\frac{\partial }{\partial r}+\frac{1}{r^{2}}\;\frac{\partial^{2}}{\partial \varphi^{2}}+\frac{\partial^{2}}{\partial z^{2}} \end{array}\right)R\left(r\right)\;\Phi \left(\varphi \right)\;Z\left(z\right).$$

#### Appendix C.1.1. Separation of the Laplace Equation

Applying the Laplace operator and dividing by R(r)Φ(φ)Z(z) yields

(A33) $$0=\frac{1}{r\;R(r)}\frac{\partial }{\partial r}r\frac{\partial }{\partial r}R\left(r\right)+\frac{1}{r^{2}\;\Phi \left(\varphi \right)}\;\frac{\partial^{2}}{\partial \varphi^{2}}\Phi \left(\varphi \right)+\frac{1}{Z(z)}\frac{\partial^{2}}{\partial z^{2}}Z\left(z\right).$$

The first and second term depend on *r* and φ whereas the third term only depends on height *z*. (A33) can only hold if both parts are constant. Therefore, (A33) separates into an ordinary differential equation

(A34) $$\frac{1}{Z(z)}\frac{\partial^{2}}{\partial z^{2}}Z\left(z\right)-k^{2}=0$$

for the height and, after multiplication by $r^{2}$, into

(A35) $$0=\frac{r}{R(r)}\frac{\partial }{\partial r}r\frac{\partial }{\partial r}R\left(r\right)+k^{2}\;r^{2}+\frac{1}{\Phi (\varphi )}\;\frac{\partial^{2}}{\partial \varphi^{2}}\Phi \left(\varphi \right)$$

for the radial and azimuth part. Again, both parts must be constant, which separates (A35) into an azimuth part

(A36) $$\frac{1}{\Phi (\varphi )}\;\frac{\partial^{2}}{\partial \varphi^{2}}\Phi \left(\varphi \right)+m^{2}=0$$

and a radial part

(A37) $$r\;\frac{\partial }{\partial r}r\frac{\partial }{\partial r}R\left(r\right)+\left(k^{2}\;r^{2}-m^{2}\right)R\left(r\right)=0.$$

#### Appendix C.1.2. Solution of the Azimuth Part

A solution to (A36) is

(A38) $$\Phi_{m}\left(\varphi \right)=A_{m}\;\cos\left(m\varphi \right)+B_{m}\;\sin\left(m\varphi \right).$$

This solution must be periodic with a period of 2π. Thus, *m* must be an integer value.

#### Appendix C.1.3. Solution of the Height Part

A solution for (A34) is

(A39) $$Z_{k}\left(z\right)=\left\{\begin{array}{cc} C_{k}\;\exp\left(k\;z\right)+D_{k}\;\exp\left(-k\;z\right),\; & k\neq 0,\\ C_{0}+D_{0}\;z,\; & k=0. \end{array}\right.$$

In general, *k* is non-integer. So the coefficients $C_{k}$ and $D_{k}$ are functions dependent on *k*. The solution above considers the special case k=0. This leads to magnetic strength fields which do not depend on *z*.

#### Appendix C.1.4. Solution of the Radial Part

Substituting x=kr in (A37) leads to the well-known Bessel differential equation

(A40) $$x\;\frac{\partial }{\partial x}x\frac{\partial }{\partial x}R\left(x\right)+\left(x^{2}-m^{2}\right)R\left(x\right)=0.$$

A solution is the Bessel functions of the first kind

(A41) $$J_{m}\left(x\right)=\sum_{\ell =0}^{\infty }\frac{{\left(-1\right)}^{\ell }}{\Gamma (m+\ell +1)\;\ell !}\;{\left(\frac{x}{2}\right)}^{2\ell +m},$$

with Γ(x) as the Gamma function. For integer values x=n, this function is Γ(n+1)=n!. For non-integer values *m*, $J_{m}\left(x\right)$ and $J_{-m}\left(x\right)$ provide two linearly independent solutions of the Bessel differential equation. From the solution of the azimuth part we know that *m* is an integer value. For *m* being an integer, a second linear independent solution is the Bessel function of the second kind, also called Neumann function

(A42) $$N_{\nu }\left(x\right)=\frac{J_{\nu }\left(x\right)\;\cos\left(v\;x\right)-J_{-v}\left(x\right)}{\sin(\nu \;x)}.$$

For integer values *m* we have to take the limit, which yields

(A43) $$N_{m}\left(x\right)=\lim_{\nu \to m}N_{\nu }\left(x\right)=\lim_{\nu \to m}\frac{J_{\nu }\left(x\right)\;\cos\left(v\;x\right)-J_{-v}\left(x\right)}{\sin(\nu \;x)}.$$

For integer *m*, Bessel functions of the first kind, $J_{m}\left(x\right)$, are finite at the origin x=0. The Neumann functions, $N_{m}\left(x\right)$, diverge for x→0.

For the special case k=0, (A37) simplifies to

(A44) $$r\;\frac{\partial }{\partial r}r\frac{\partial }{\partial r}R\left(r\right)+m^{2}\;R\left(r\right)=0.$$

This leads to a different form of solution. However, this solution is equivalent to that which we obtain by taking the limit k→0, i.e., considering the Bessel and Neumann functions for small arguments.

In summary, the solution for the radial part in is

(A45) $$R_{k,m}\left(r\right)=\left\{\begin{array}{cc} E_{m}\;J_{m}\left(kr\right)+F_{m}\;N_{m}\left(kr\right),\; & k\neq 0,\\ E_{m}\;r^{m}+F_{m}\;\frac{1}{r^{m}},\; & k=0,m>0,\\ E_{0}+F_{0}\;\ln\left(\frac{r}{r_{0}}\right),\; & k=0,m=0. \end{array}\right.$$

#### Appendix C.1.5. The General Solution

For the general solution we combine (A38), (A39), (A45) and get

(A46) $$\Psi \left(r,\varphi ,z\right)=\sum_{m=0}^{\infty }\int_{k=0}^{\infty }\Phi_{m}\left(\varphi \right)\;Z_{k}\left(z\right)\;R_{k,m}\left(r\right)\;dk.$$

### Appendix C.2. Solution of the Boundary Value Problem

First let us consider a homogeneous outer magnetic field with arbitrary direction. Since the dimension of the cylinder in z-direction is infinite, there is no dependency of the resulting magnetic field on dimension *z*. For this reason, it is obvious that the cylinder does not influence the z-component $H_{z,\infty }\;e_{z}$ of an outer homogenous field. So, any constant magnetic strength vector field in z-direction, or equivalently a magnetic potential of form $\Psi =-H_{z,\infty }\;z$ solves the boundary value problem. A consequence of the longitudinal invariance is that the separation constant

(A47) k=0.

Therefore, we consider a homogeneous outer magnetic strength field with a direction in the x-y-plane. Without loosing generality we assume a homogeneous magnetic strength vector field in x-direction with magnitude $H_{\infty }$ which we can express as

(A48) $$H_{\infty }=H_{\infty }\;e_{x}=H_{\infty }\cos\left(\varphi \right)\;e_{r}-H_{\infty }\sin\left(\varphi \right)\;e_{\varphi }$$

in Cartesian respectively cylinder coordinates, which follows from the corresponding magnetic potential field

(A49) $$\Psi_{\infty }=-H_{\infty }\;x=-H_{\infty }\;r\;\cos\left(\varphi \right).$$

For k=0 we start with the outer magnetic potential field which has to fulfill the boundary constraint at infinite distance, $\lim_{r\to \infty }\Psi_{\mathrm{out}}\overset{!}{=}\Psi_{\infty }$ in particular

(A50) $$\begin{array}{c} \lim_{r\to \infty }E_{0}+F_{0}\;\ln\left(\frac{r}{r_{0}}\right)+\sum_{m=1}^{\infty }\left(E_{m}\;r^{m}+F_{m}\;\frac{1}{r^{m}}\right)\;\left(A_{m}\;\cos\left(m\;\varphi \right)+B_{m}\;\sin\left(m\;\varphi \right)\right)\\ \overset{!}{=}-H_{\infty }\;r\;\cos\left(\varphi \right). \end{array}$$

Comparing both sides of the equation we observe that $E_{m}=0$ for m≠1, $E_{1}=-H_{\infty }$, $F_{0}=0$ and $B_{m}=0$, which provides the remaining outer field as

(A51) $$\Psi_{\mathrm{out}}=-H_{\infty }\;r\;\cos\left(\varphi \right)+\sum_{m=1}^{\infty }F_{m}\;\frac{1}{r^{m}}\;\;\cos\left(m\;\varphi \right).$$

The inner potential field requires $F_{m}=0$. Otherwise the field diverges for r→0. From the outer field we observe that there are no sin(·) components. Therefore, these terms will also vanish from the inner solution. This remaining potential field is

(A52) $$\Psi_{\mathrm{in}}=\sum_{m=1}^{\infty }{\tilde{A}}_{m}\;r^{m}\;\cos\left(m\;\varphi \right),$$

where the coefficients $E_{m}$ have been included in ${\tilde{A}}_{m}$.

For m≥2 the boundary conditions at the cylinder’s surface $\left(r=r_{\mathrm{cyl}},0\leq \varphi <2\pi ,-\infty \leq z\leq +\infty \right)$ from (5) respectively (6) yield the equation system

(A53) $$\begin{array}{cccc} m\;r_{\mathrm{cyl}}^{m-1}\;{\tilde{A}}_{m} & - & \frac{m}{r_{\mathrm{cyl}}^{m+1}}\;F_{m} & =0\\ \mu_{\mathrm{in}}\;m\;r_{\mathrm{cyl}}^{m-1}\;{\tilde{A}}_{m} & + & \frac{\mu_{\mathrm{out}}\;m}{r_{\mathrm{cyl}}^{m+1}}\;F_{m}\; & =0 \end{array}$$

This system of equation has full rank and, therefore, yields to the trivial solution ${\tilde{A}}_{m}=0$ and $F_{m}=0$ only. For m=1, however, $E_{1}=-H_{\infty }$. After some simplifications we obtain

(A54) $$\begin{array}{ccccc} {\tilde{A}}_{1} & - & \frac{1}{r_{\mathrm{cyl}}^{2}}\;F_{1}\; & =\; & -H_{\infty }\\ \frac{\mu_{\mathrm{in}}}{\mu_{\mathrm{out}}}\;{\tilde{A}}_{1} & + & \frac{1}{r_{\mathrm{cyl}}^{2}}\;F_{1}\; & =\; & -H_{\infty } \end{array}$$

Solving the equations above provides

(A55) $$\begin{array}{cccc} {\tilde{A}}_{1} & =-H_{\infty }\;\frac{2}{\frac{\mu_{\mathrm{in}}}{\mu_{\mathrm{out}}}+1}\; & F_{1} & =H_{\infty }\frac{\frac{\mu_{\mathrm{in}}}{\mu_{\mathrm{out}}}-1}{\frac{\mu_{\mathrm{in}}}{\mu_{\mathrm{out}}}+1}\;r_{\mathrm{cyl}}^{2} \end{array}$$

and leads to the scalar magnetic potential fields

(A56) $$\begin{array}{cc} \Psi_{\mathrm{in}} & =-H_{\infty }\;\;\frac{2}{\frac{\mu_{\mathrm{in}}}{\mu_{\mathrm{out}}}+1}\;r\;\cos\left(\varphi \right) \end{array}$$

(A57) $$\begin{array}{cc} \Psi_{\mathrm{out}}\; & =H_{\infty }\left(\frac{\frac{\mu_{\mathrm{in}}}{\mu_{\mathrm{out}}}-1}{\frac{\mu_{\mathrm{in}}}{\mu_{\mathrm{out}}}+1}{\left(\frac{r_{\mathrm{cyl}}}{r}\right)}^{2}-1\right)\;r\;\cos\left(\varphi \right) \end{array}$$

inside and outside the cylinder.
